# Bone-targeting delivery of platelet lysate exosomes ameliorates glucocorticoid-induced osteoporosis by enhancing bone-vessel coupling

**DOI:** 10.1186/s12951-022-01400-1

**Published:** 2022-10-31

**Authors:** Gang Zheng, Hai-Wei Ma, Guang-Heng Xiang, Gao-Lu He, Han-Chen Cai, Zi-Han Dai, Yan-Lin Chen, Yan Lin, Hua-Zi Xu, Wen-Fei Ni, Cong Xu, Hai-Xiao Liu, Xiang-Yang Wang

**Affiliations:** 1 Key Laboratory of Orthopaedics of Zhejiang Province, Department of Orthopaedics, The Second Affiliated Hospital and Yuying Children’s Hospital of Wenzhou Medical University, Wenzhou, 325000 Zhejiang Province China; 2 The Second School of Medicine, Wenzhou Medical University, Wenzhou, 325000 Zhejiang Province China; 3 Department of Orthopaedic Surgery, Lishui Central Hospital and Fifth Affiliated Hospital of Wenzhou Medical University, Lishui, 323000 Zhejiang Province China

**Keywords:** Glucocorticoid, Osteoporosis, Platelet lysate, Exosome, Bone-targeting

## Abstract

**Background:**

Glucocorticoids (GCs) overuse is associated with decreased bone mass and osseous vasculature destruction, leading to severe osteoporosis. Platelet lysates (PL) as a pool of growth factors (GFs) were widely used in local bone repair by its potent pro-regeneration and pro-angiogenesis. However, it is still seldom applied for treating systemic osteopathia due to the lack of a suitable delivery strategy. The non-targeted distribution of GFs might cause tumorigenesis in other organs.

**Results:**

In this study, PL-derived exosomes (PL-exo) were isolated to enrich the platelet-derived GFs, followed by conjugating with alendronate (ALN) grafted PEGylated phospholipid (DSPE-PEG-ALN) to establish a bone-targeting PL-exo (PL-exo-ALN). The in vitro hydroxyapatite binding affinity and in vivo bone targeting aggregation of PL-exo were significantly enhanced after ALN modification. Besides directly modulating the osteogenic and angiogenic differentiation of bone marrow mesenchymal stem cells (BMSCs) and endothelial progenitor cells (EPCs), respectively, PL-exo-ALN also facilitate their coupling under GCs’ stimulation. Additionally, intravenous injection of PL-exo-ALN could successfully rescue GCs induced osteoporosis (GIOP) in vivo.

**Conclusions:**

PL-exo-ALN may be utilized as a novel nanoplatform for precise infusion of GFs to bone sites and exerts promising therapeutic potential for GIOP.

**Graphical Abstract:**

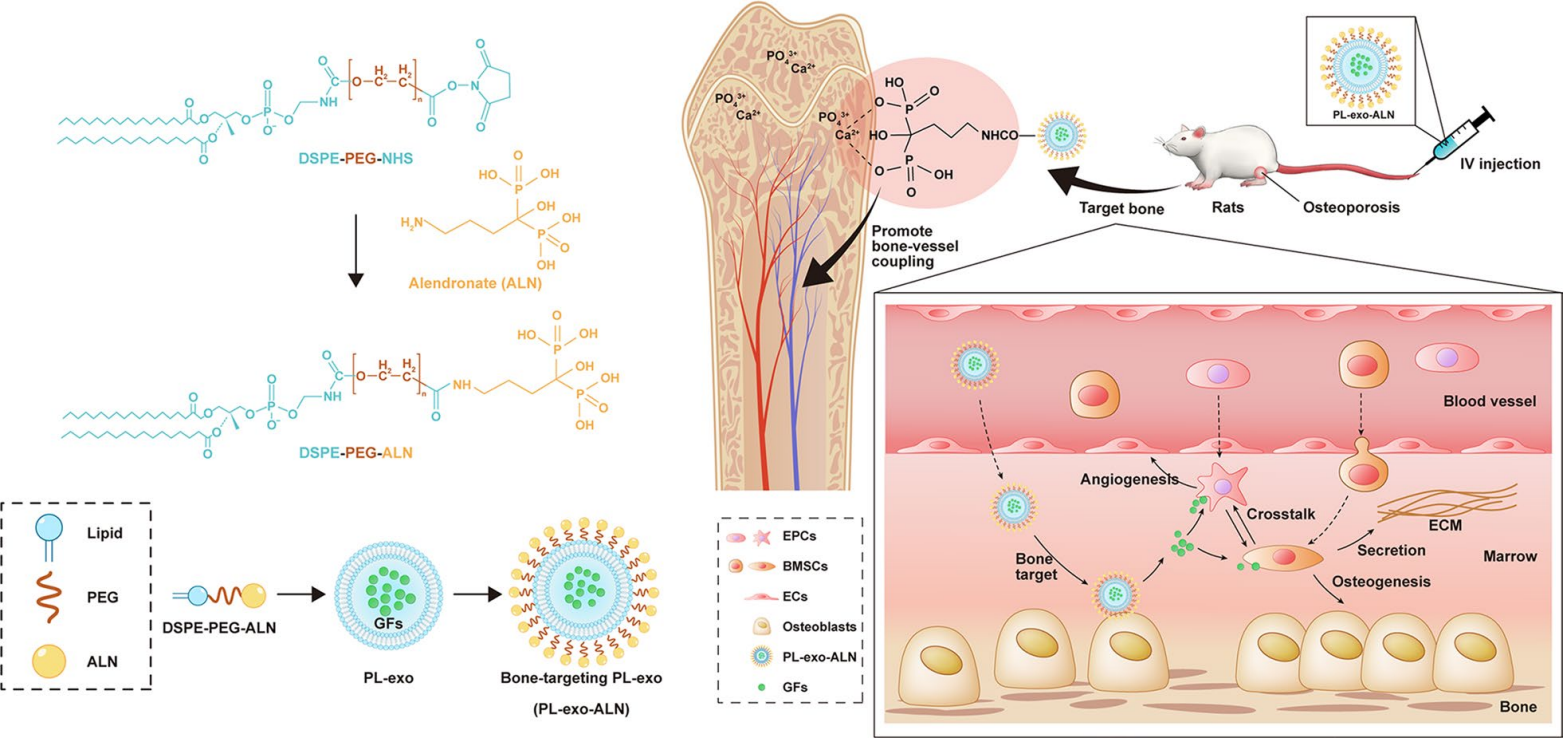

**Supplementary Information:**

The online version contains supplementary material available at 10.1186/s12951-022-01400-1.

## Introduction

Glucocorticoids (GCs) are routinely applied as an anti-inflammatory and immunosuppressive agent to treat inflammatory and autoimmune diseases such as rheumatoid arthritis and systemic lupus erythematosus [1]. Unfortunately, long-term abuse of GCs commonly results in a decline in bone mineral density and dramatic bone loss, especially in cancellous bone, thereby increasing the risk of fragility fractures and even deteriorating osteonecrosis [2]. This kind of secondary osteoporosis was termed glucocorticoid-induced osteoporosis (GIOP). Unlike age- or menopause-related osteoporosis occurring mostly in old patients, GIOP is the most common form of pediatric osteoporosis with 29–45% prevalence in children, which brought a heavy social and economic burden [3]. Thus, a safe and effective therapeutic strategy for the management of GIOP is of great urgency and significance.

The exact pathophysiology of GIOP is rather complex and has not yet been entirely revealed [4]. Long-term exposure of GCs comprehensively destroys the microstructure of bone by osteoclast activation, osteoinhibition, osteocyte apoptosis and endothelial cell damage. But current mainstream views believed that GCs-medicated persistent and severe inhibition of osteogenesis and impediment of intraosseous vasculature formation is the main culprits of GIOP [5]. Therefore, enhancing osteogenesis and angiogenesis coupling is highly desirable for GIOP. Previous evidence showed that endogenous platelet-derived growth factor type BB (PDGF-BB) is the crucial regulator responsible for recruiting endothelial and mesenchymal progenitor cells to couple angiogenesis and osteogenesis [6–8]. It, along with other growth factors (GFs) like vascular endothelial growth factor (VEGF), insulin-like growth factor-1 (IGF-1), et al., are decreased in GIOP, resulting in the decreased type H vessels and inhibiting osteogenesis [2, 9, 10]. However, whether exogenous replenishment of GFs into bone tissue could ameliorate GIOP is still not been reported.

Platelet lysates (PL), as an economic cocktail of GFs, mainly consists of PDGF, VEGF, transforming growth factor-β (TGF-β), and basic fibroblasts growth factor (bFGF), which can be easily obtained by repeated freezing-thawing circles of platelet-rich plasma (PRP) [11]. Being rich in the multiple GFs, PL exerts the promising potential in bone tissue engineering, neurogenesis, and wound repair [12, 13]. Benefiting from the removal of platelet debris, PL possesses lower immunogenicity than PRP; but as a GFs complex, the rapid leakage, short half-life, denaturation, and injection risks have essentially limited the large-scale application of PL [14–16]. The current PL-based biomaterials for tissue regeneration mainly center on PL-mixed hydrogels and PL-loaded scaffolds, which appear to be a promising substitute for synthetic GFs [17, 18]. Nevertheless, the above materials are limited to local delivery of PL and the burst releasing proteins in vivo [19]. As for systemic osteopathia such as GIOP; these materials are not minimally invasive enough nor practical for the whole skeleton. Although systemic improvement of osteogenic potentials was seen in animal models by intravenous injection of GFs, this administration strategy is still highly restricted in clinical practice due to the blind targets, denaturation, dose control difficulties, and the possible risk of thrombosis and tumorigenesis [20]. Hence, developing bone-targeted drug delivery systems for GFs is highly desirable for GIOP.

Actually, by encapsulating PL with targeted nanoparticles, PL can be specifically transported to designated tissues and minimizing its side effects. However, due to the loss of encapsulation efficiency and the toxicity and degradability of materials, this method is not the best choice for PL targeted delivery. Recently, Torreggiani et al. implied that platelet-derived exosomes help to enrich the GFs in natural nanovesicles with a high concentration and might be potent effectors of PL's property [19]. Evidence suggested that these exosomes, isolated from platelet derivatives (PRP or PL), play a beneficial role in wound healing, muscle injury, osteoarthritis, and GC-induced osteonecrosis [21–23]. According to the proteomics identification and cellular component analysis, Tang et al. demonstrated that 74.7% of PL proteins originated from the extracellular exosome, which is conducive to efficient endocytosis and maintenance of active molecules [11]. Except for being responsible for intercellular communication, exosomes as nanovesicles (50–150 nm in diameter) also represent optimal carriers for gene and drug nano-delivery; because of their large loading capacity, cargo protection, low immunogenicity and stability [24, 25]. More importantly, modifying the lipid bilayer of exosomes by targeting peptides or molecule grafted phospholipid polymer imparts targeting ability of exosomes, such as DSPE-PEG-RGD and DSPE-PEG-Folic Acid (FA) for exosomal precision medicine in oncology [26]. Therefore, platelet-derived exosomes appear to be a promising alternative to targeted GFs delivery.

Alendronate (ALN), as FDA-approved bisphosphonate for the treatment of osteoporosis, has an advantageous affinity to the bone surface, cost-effectiveness and pro-osteogenic abilities, comparing to the oligopeptides like hexapeptide and aspartic acid octapeptide [27–29]; therefore, it has been widely studied in bone-targeted drug delivery [30]. In this work, we surface modified the PL-derived exosomes (PL-exo) with ALN conjugated PEGylated phospholipid (DSPE-PEG-ALN). We termed these modified exosomes PL-exo-ALN, and found that PL-exo-ALN could be effectively accumulated in the bone surface. Up to now, this is the first study to modify PL-exo with bone-targeting ability to enhance the efficiency of GFs' delivery in bone site, and systemically assessing their therapeutic effects on GCs-inhibited osteogenesis and angiogenesis both in vitro and in vivo. We believe that our study provides new insights and future directions for using the multifunctional exosome platforms to realize targeted and precision nanomedicine in systemic bone disease.

## Results

### Preparation and characterization of ALN-conjugated PL-exosomes

**Fig. 1:**
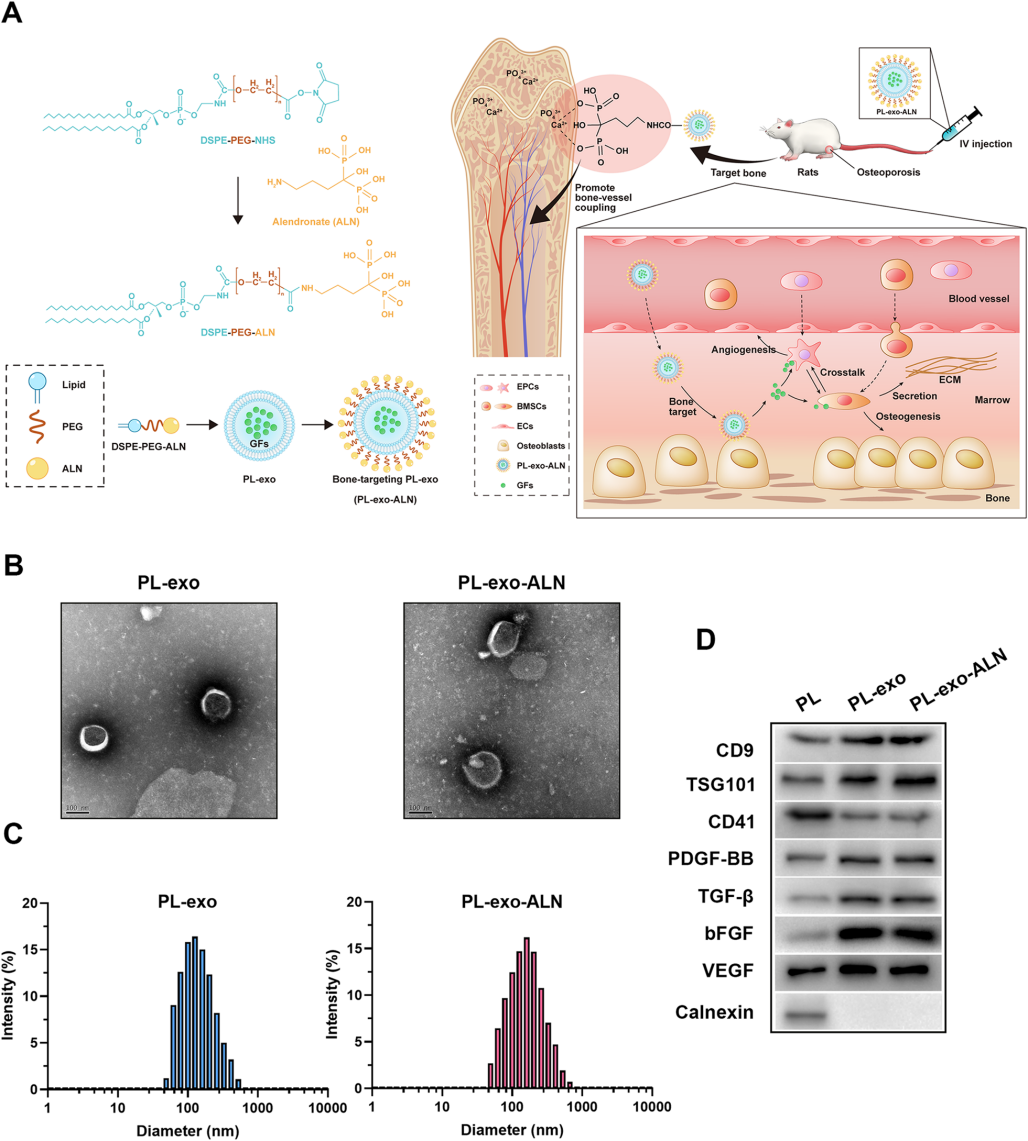
Characteristics of the isolated PL-Exos. **A** Schematic illustration of PL-exo-ALN preparation and its bone targeting mechanism. **B** Representative TEM image of PL-exo and PL-exo-ALN (scale bar: 100 nm). **C** Representative DLS results showing the size distribution of PL-exo and PL-exo-ALN. **D** Confirmation of the presence of exosomal marker proteins (CD9 and TSG101), the decrease of platelet marker (CD41), the enrich of representative GFs (PDGF-BB, TGF-β, bFGF and VEGF) and the absence of the non-exosomal protein (calnexin) by Western blot analysis

To endow PL-derived exosomes with bone-targeting ability, we fabricated DSPE-PEG-ALN by carbodiimide reaction and inserted them into the lipid bilayer of exosomes. The specific reaction route and targeting mechanism are shown in Fig. 1A. By replacing the N-terminal with DSPE-PEG-NHS, ALN was linked to the active ester via a peptide bond. And we performed the FTIR and ^1^H-NMR spectroscopy to confirm the chemical structure of ALN, DSPE-PEG-NHS and synthesized DSPE-PEG-ALN. As shown in Additional file 1: Fig. S1, elimination of the peak at 1733 cm^−1^ (characteristic peak of NHS group) was observed in DSPE-PEG-ALN compared to DSPE-PEG-NHS, which was related to the nucleophilic attack of the amine groups of ALN on the NHS-activated carbonyl group from DSPE-PEG-NHS. According to the ^1^H-NMR results, the DSPE-PEG-ALN group revealed a reduction of a signal of singlet at 2.67 (NHS group) and the appearance of ALN characteristic signal relative to the DSPE-PEG-NHS (Additional file 1: Figs. S2–S4). ALN showed the characteristic signal at 1.88 and 2.93 ppm corresponding to CH_2_ protons, but this signal was shifted to 1.98 and 3.13 ppm in the spectrum of DSPE-PEG-ALN (Additional file 1: Figs. S2 and S4). These findings suggested the successful synthesis of DSPE-PEG-ALN.

Then, we performed the TEM, DLS analysis, zeta-potential and western blotting to characterize the morphology, particle size and protein composition of PL-exo and PL-exo-ALN. TEM visually displayed the typical cup-shaped morphology of two exosomes with a size below 150 nm (Fig. 1B). According to DLS analysis, the particle sizes of these two exosomes ranged from 30 to 200 nm but mainly concentrated around 150 nm, which was consistent with the TEM results (Fig. 1C). Compared to the PL, PL-derived exosomes possess lower zeta-potential, but ALN functionalization also modestly altered zeta-potential from  − 16.8 mV for PL-exo to − 14.6 mV for PL-exo-ALN (Additional file 1: Fig. S6A). Additionally, western blotting showed that PL-exo and PL-exo-ALN were positive for exosomal surface markers CD9 and TSG101(Fig. 1D). As for the platelet markers, the expression level of CD41 in these two exosome groups was lower than that in the native PL group; especially the endoplasmic reticulum membrane protein calnexin was also negative in exosome groups (Fig. 1D), which is abundant in platelets but not exist in exosomes [23]. The protein level of PDGF-BB, TGF-β, bFGF and VEGF were upregulated in PL-exo and PL-exo-ALN (Fig. 1D). Meanwhile, ELISA results found higher levels of PDGF-BB, TGF-β, bFGF, VEGF and PDGF-AB in PL-exo relative to PL, which indicated that PL derived exosomes contribute to the enrichment and carrying of GFs (Additional file 1: Figure S6D). These data confirmed these isolated vesicles and further processed nanoparticles belonged to PL-derived exosomes.

### In vitro and in vivo bone-targeting ability of PL-exo-ALN

**Fig. 2:**
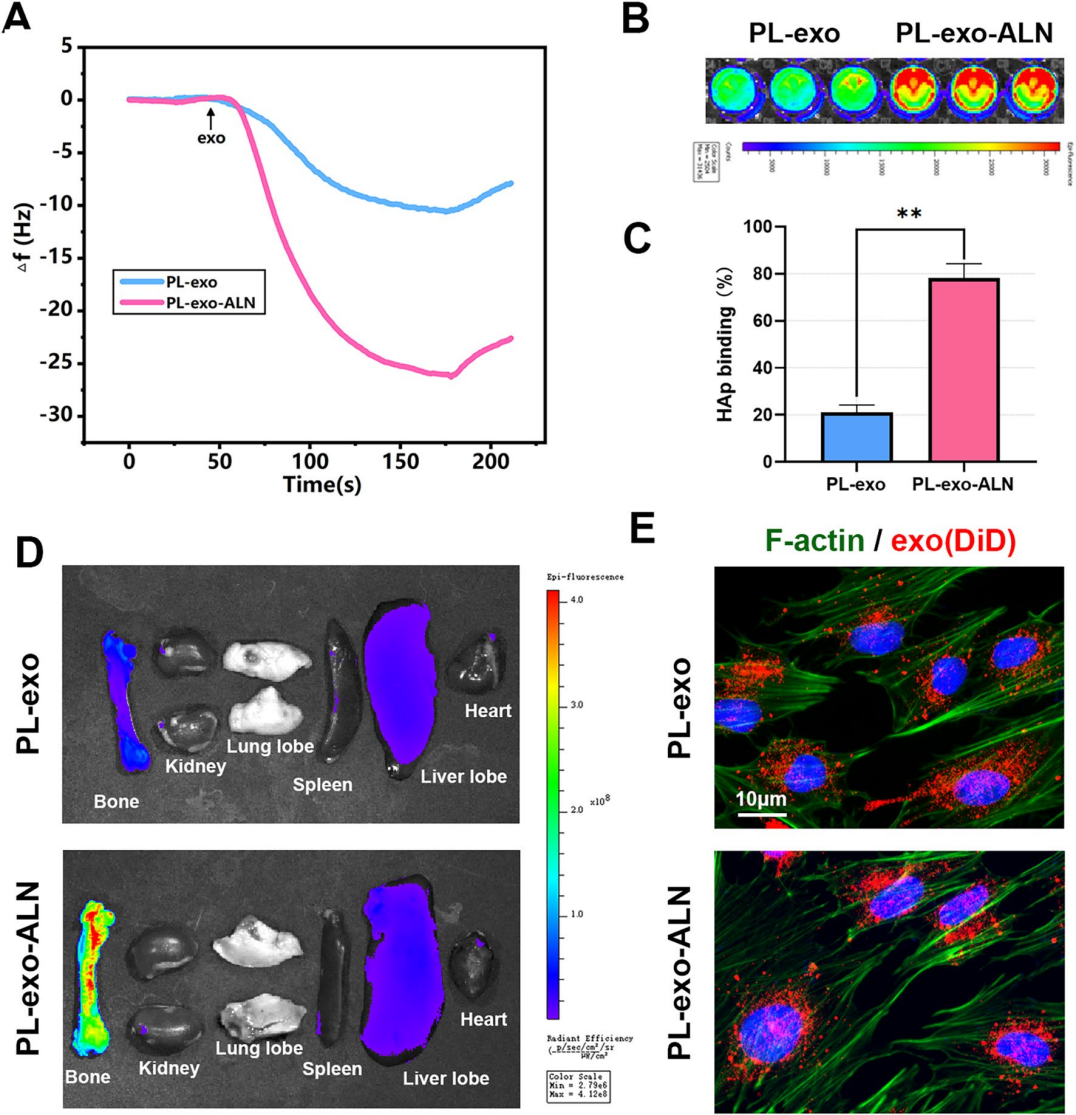
Bone-targeting ability of PL-exo-ALN. **A** Frequency shifts of exosomes deposition on the HAp coated sensor were detected by QCM-D. **B** Fluorescent Image of DiD-labelled exosomes binding to HAp were detected by IVIS. **C** Quantitative analysis of DiD-labelled exosomes binding to HAp by a fluorescence microplate reader. **D** Fluorescent Image of the in vivo distribution of DiD-labelled exosomes by IVIS. **E** Internalization of exosomes into BMSCs (scale bar: 10 μm). All results are presented as the means ± SDs, ^*^P < 0.05, ^**^P < 0.01

As a bisphosphonate approved by the FDA for osteoporosis treatment, the two phosphate groups of ALN possess a high affinity for abundant HAp in the bone microenvironment, thereby achieving bone-targeted delivery. To dynamically assess the HAp binding capacity of modified exosomes, QCM-D with HAp coated sensor was applied to mimic the bone surface, which is a nanogram-scale microbalance utilizing the inverse piezoelectric effect to measure mass adsorption and changes in the fluid. The frequency shift is positively correlated with the deposition of exosomes on the sensor surface. As shown in Fig. 2A, the Δf value of PL-exo-ALN showed a larger and faster decrease than PL-exo, indicating that Aln modification promoted the binding of exosomes to HAp. Moreover, we incubated DiD-labeled exosomes with HAp particles and detected the difference of liquid fluorescence intensity before and after incubation to quantify the HAp binding ability of exosomes. According to in vitro IVIS results, PL-exo-ALN group showed higher fluorescent intensity than the PL-exo group (Fig. 2B). Quantitative analysis by fluorescence spectrophotometer found that after the modification of Aln, the binding affinity of PL-derived exosomes to HAp increased from 21 to 78% (Fig. 2C). As for the biodistribution of exosomes in vivo, IVIS images revealed that the DiD signal was dominantly distributed in liver and bone, and Aln-modified PL-exo were more likely to be enriched in bone marrow (Fig. 2D). Finally, we evaluated the uptake ability of BMSC cells to the two exosomes by co-culturing DiD-labeled exosomes and BMSC cells. Interestingly, unlike the combination of HAp, exosome internalization results revealed the similar endocytic ability of BMSCs to these two kinds of exosomes, which indicated that Aln modification helps exosome to bind the bone minerals instead of BMSCs (Fig. 2E).

### PL-exo-ALN reversed Dex inhibited-osteogenic differentiation of BMSCs in vitro

**Fig. 3:**
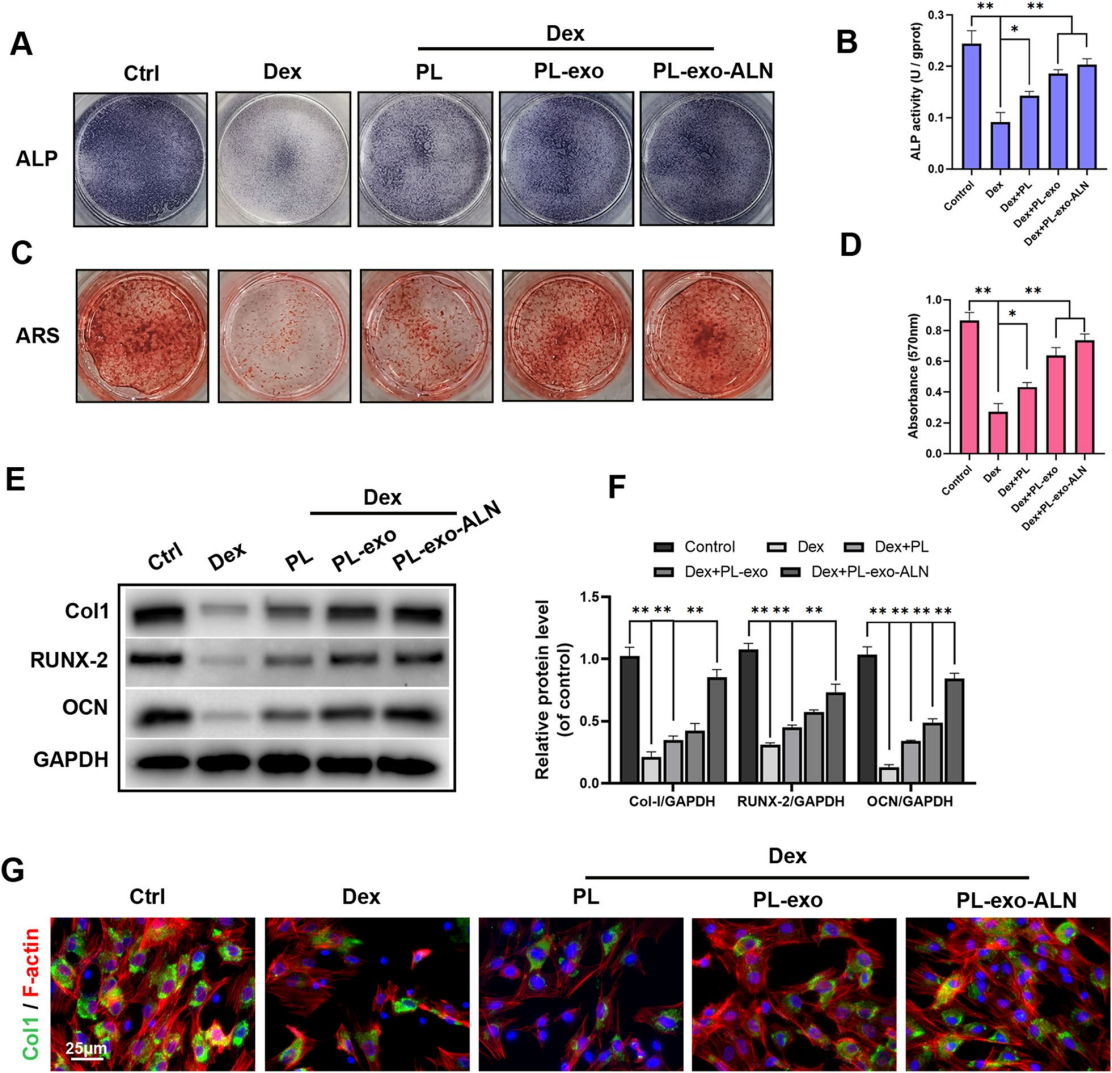
Effects of PL, PL-exo and PL-exo-ALN on Dex-induced osteogenesis inhibition in BMSCs. **A**–**B** Early osteogenic differentiation was determined by ALP staining and ALP activity assays after 5 days of induction. **C**–**D** Late osteogenic differentiation was determined by Alizarin Red staining and the calcium deposition was quantified by measuring the optical density, after 14 days of induction. **E**–**F** The expression levels of specific proteins in BMSCs treated as indicated for 3 days. **G** Immunofluorescence staining of Col-I (green), F-actin(red) and nucleus (blue) after 3 days induction (scale bar: 25 μm). All results are presented as the means ± SDs, ^*^P < 0.05, ^**^P < 0.01

GCs exposure mediate cellular apoptosis and promote the preferential differentiation of BMSCs into adipocytes rather than osteoblasts. To evaluate the effect of PL and its exosomes on the Dex-treated BMSCs in vitro, CCK-8, ALP activity and calcium deposition were detected and quantified at 3th day of culture or 5 and 14 days after osteoinduction. Base on the CCK-8 result, we found that Dex stimulation could inhibit the proliferation of BMSCs, but this phenomenon was reversed after treatment with PL or PL-derived exosomes (Additional file 1: Fig. S6B). Notably, PL-exo or PL-exo-ALN addition could obviously up-regulate the cellular viability compared to PL group. According to ALP and ARS staining of BMSCs, Dex treatment inhibited ALP expression and activity and mineralized nodule formation. Nevertheless, this phenomenon was partially reversed by PL treatment, and was obviously rescued after PL-exo and PL-exo-ALN treatment (Fig. 3A–D). Furthermore, the level of osteogenic-related proteins like RUNX2, COL1 and OCN were detected by western blotting. The results showed the protein levels of RUNX2, COL1 and OCN were all down-regulated after Dex exposure, but the administration of PL and PL-derived exosomes increased these proteins' expression, especially in the PL-exo-ALN group (Fig. 3E–F). Similar results were also found in Collagen-I immunofluorescent staining (Fig. 3G).

### PL-exo-ALN rescued Dex inhibited-angiogenesis of EPCs in vitro

**Fig. 4:**
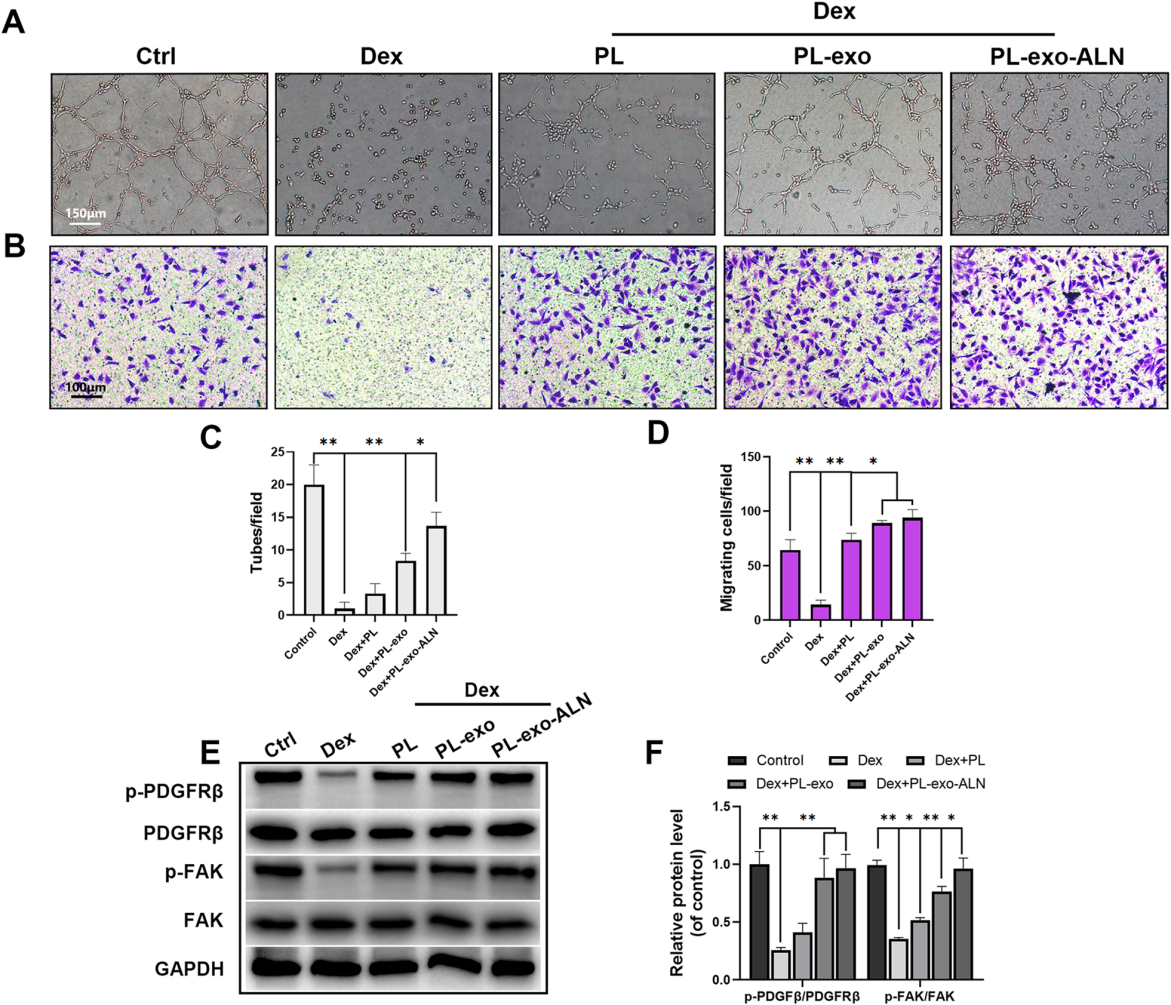
Effects of PL, PL-exo and PL-exo-ALN on Dex-induced angiogenesis inhibition in EPCs. **A** In vitro tube formation assay of EPCs treated as indicated. (scale bar: 150 μm). **B** The migration evaluation of EPCs treated as indicated by transwell assay (scale bar: 100 μm). **C** Quantification of tube formation. **D** Quantification of migrated cells. **E**–**F** The phosphorylation levels of PDGFRβ and FAK in EPCs treated as indicated for 24 h. All results are presented as the means ± SDs, ^*^P < 0.05, ^**^P < 0.01

Apart from affecting osteogenic differentiation, combating intra-osseous angiogenesis is a crucial internal factor for GIOP, which is achieved by reducing endogenous growth factors and mediating oxidative damage to the vascular endothelium. Therefore, endothelial migration and tube formation experiments were performed to evaluate the angiogenesis ability of PL and PL exosomes on Dex stimulated EPCs. As shown in Fig. 4A–D, Dex treatment significantly prevented the nodes and loop structures formation of EPCs and decreased the cellular migration mobility. However, PL, PL-exo and PL-exo-ALN intervention significantly reversed these inhibitory effects. Although no significant difference was displayed between PL-exo and PL-exo-ALN groups, these two exosomes showed better pro-angiogenic and pro-migrative abilities than native PL group (Fig. 4A–D). As an essential factor in PDGF-BB-mediated H-type vascular development in bone, the PDGFRβ/FAK signal was found to be inactivated in GCs-mediated osteoporosis [9]. By western blot analysis, the phosphorylated level of PDGFRβ and FAK were significantly decreased after Dex intervention, but they were restored by PL, PL-exo and PL-exo-ALN treatment, especially in exosomes groups (Fig. 4E–F). These data suggested that the PL-derived exosomes mediated pro-angiogenic effects might be related to PDGF-BB/PDGFRβ/FAK axis.

Notably, under physiological conditions, PL-exo treatment alone could directly promote the osteogenic and angiogenic differentiation of BMSCs and EPCs (Additional file 1: Fig. S7A–E). β-catenin and Hif-α, as crucial transcription factors, could mediate the expression of osteogenesis- and angiogenesis-related proteins. AKT is a signal-transmission molecule with multiple cellular funcitons including cell proliferation, migration and differentiation [31, 32]. To further explore the underlying mechanism of PL-exo, we evaluated the activity of AKT/β-catenin in BMSCs and AKT/Hif-α axis in EPCs, respectively. As shown in Additional file 1: Figure S7F–I, the nuclear expression levels of β-catenin and Hif-α, and the phosphorylation level of AKT were elevated in PL-exo-treated BMSCs and EPCs, respectively. But inhibition of AKT phosphorylation by MK-2206 reversed nuclear transcription of β-catenin and Hif-α, and PL-exo-mediated expression of Col1 and VEGF (Additional file 1: Fig. S7F–I). PL and PRP-derived exosomes could activate β-catenin and Hif-α through phosphorylation of ERK1/2 and AKT signaling to trigger osteogenesis and angiogenesis, which is basically consistent with our results [11, 23]. Taken together, these data indicated that AKT/β-catenin and AKT/ Hif-α axis involve in the pro-osteogenic and pro-angiogenic effects of PL-exo.

### PL-exo-ALN promoted the crosstalk between BMSCs and EPCs under GCs stimulation

**Fig. 5:**
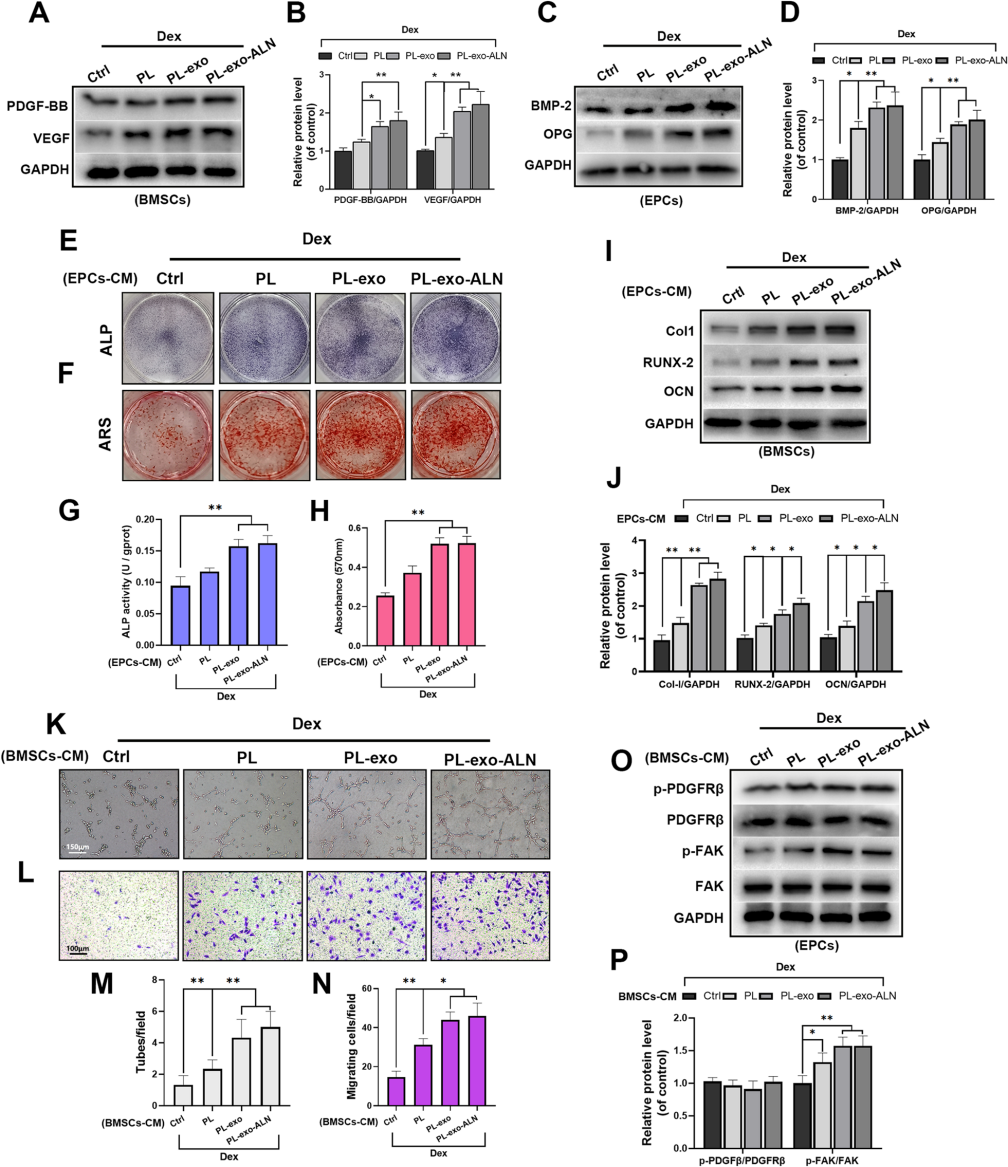
The cross-talk between BMSCs and EPCs under the Dex-stimulated condition after treatment. **A**–**B** The levels of PDGF-BB and VEGF in Dex-stimulated BMSCs treated as indicated for 24 h. **C**–**D** The levels of BMP-2 and OPG in Dex-stimulated EPCs treated as indicated for 3 days. **E**, **G** Early osteogenic differentiation of BMSCs was determined by ALP staining and ALP activity assays after 5 days of induction. **F**, **H** Late osteogenic differentiation of BMSCs was determined by Alizarin Red staining and the calcium deposition was quantified by measuring the optical density, after 14 days of induction. **I**–**J** The expression levels of specific proteins in BMSCs treated as indicated for 3 days. **K** In vitro tube formation assay of EPCs treated as indicated (scale bar: 150 μm). **L** The migration evaluation of EPCs treated as indicated by transwell assay (scale bar: 100 μm). **M** Quantification of tube formation. **N** Quantification of migrated cells. **O**–**P** The phosphorylation levels of PDGFRβ and FAK in EPCs treated as indicated for 24 h. All results are presented as the means ± SDs, ^*^P < 0.05, ^**^P < 0.01

Interestingly, BMSCs and EPCs do not function independently, but through secreting endogenous GFs or proteins to communicate with each other to participate in the recruitment, proliferation and differentiation of each other [7, 33]. PDGF-BB and VEGF from BMSCs as well as BMP-2 and OPG in EPCs were reported to be responsible for potential osteogenic-angiogenic coupling [34, 35]. To further check, we first detected the effects of PL and PL-derived exosomes on the above factors in Dex-treated BMSCs and EPCs. The results showed that compared with the Dex-stimulated BMSCs group, PL, PL-exo and PL-exo-ALN up-regulated the PDGF-BB and VEGF levels of BMSCs, especially in exosomes treated conditional medium (Fig. 5A–B). Whereas the levels of BMP-2 and OPG in EPCs were significantly increased relative to those treated with Dex alone, after PL, PL-exo and PL-exo-ALN treated BMSCs’ conditional medium incubation (Fig. 5C–D). Furthermore, as is presented in Fig. 5E–J, the ALP activity, calcium deposition potential and the protein level of osteogenic markers (Col-I, Runx-2, and OCN) of BMSCs under Dex exposure were all enhanced by incubating with PL, PL-exo and PL-exo-ALN treated EPCs’ conditional medium. On the other hand, the Dex-damaged angiogenic and migrative abilities of EPCs were also reversed by culturing in PL, PL-exo and PL-exo-ALN pretreated BMSCs’ conditional medium (Fig. 5K–N). In terms of PDGFRβ/FAK signaling, PL and PL-exos pretreated BMSCs’ conditional medium increased the phosphorylation level of FAK, but did not affect the PDGFRβ activity in Dex-treated EPCs (Fig. 5O–P). Taken together, these data suggest except for direct modulating the differentiation of BMSCs and EPCs, PL, PL-exo and PL-exo-ALN also could facilitate the osteogenic-angiogenic coupling.

### PL-exo-ALN ameliorated GIOP in a rat model by both enhancing bone anabolism and angiogenesis

**Fig. 6:**
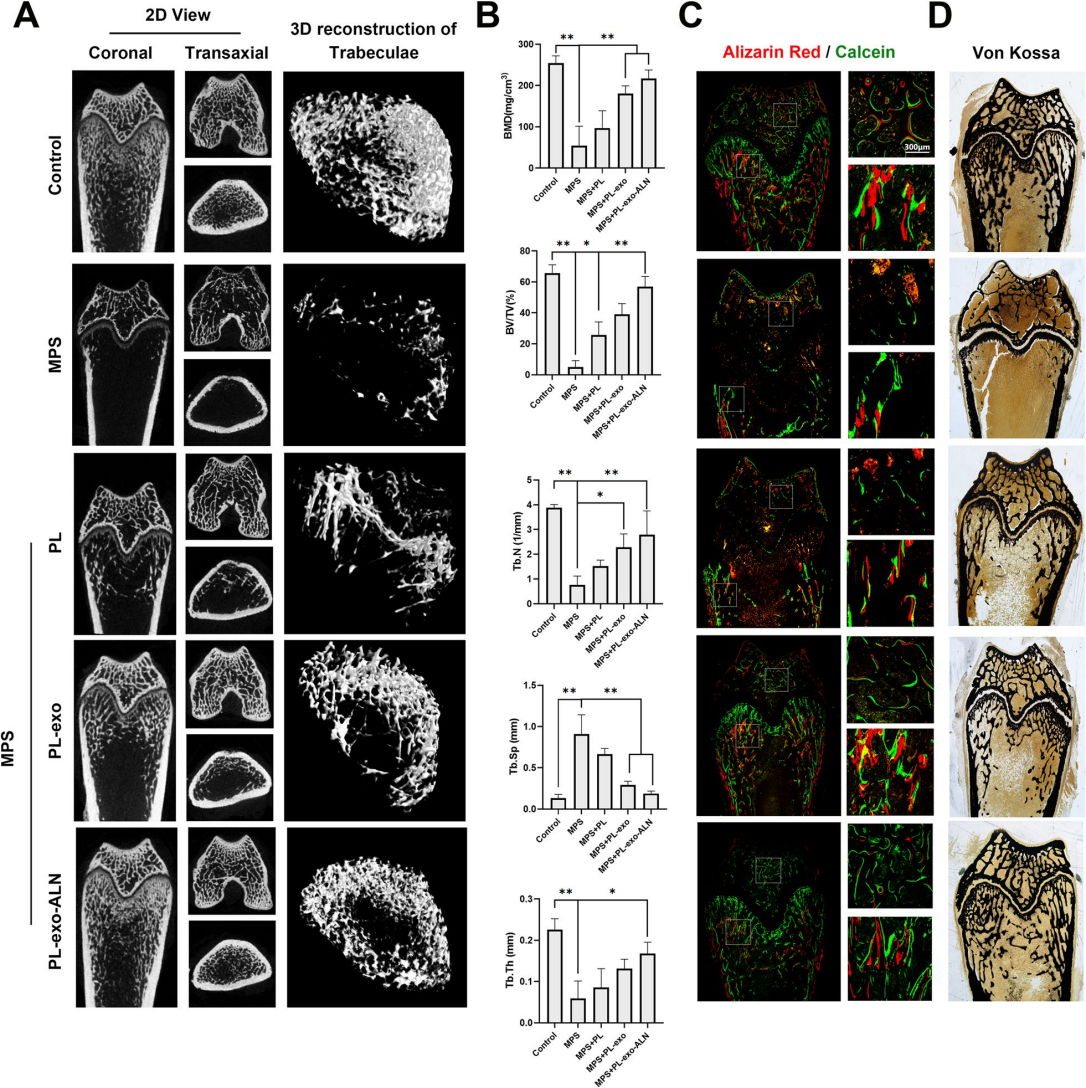
Effects of PL, PL-exo and PL-exo-ALN on MPS-induced bone mass loss in vivo. **A** 2D and 3D reconstructive μ-CT images in the distal femurs of five groups. **B** Quantification of the bone mineral density (BMD), trabecular bone volume fraction (BV/TV), trabecular number (Tb.N), trabecular thickness (Tb.Th) and trabecular separation (Tb.Sp) by CTAn of five groups. **C** Images of dynamic bone formation with different treatments were monitored the fluorochrome labeling of five groups (scale bar: 300 μm). **D** Bone minerals of different groups were examined by Von Kossa staining of five groups. All results are presented as the means ± SDs, ^*^P < 0.05, ^**^P < 0.01

To clarify the in vivo effects of PL, PL-exo and PL-exo-ALN on GCs induced bone mass loss, we created a rat GIOP model by intramuscular injection of MPS. And micro-CT scanning was carried out first to estimate and quantify the trabecular bone at the metaphysis of distal femur below the growth plate as a region of interest (ROI) (Fig. 6A, B). By the 3D reconstruction of trabeculae, it was intuitively found that long-term exposure with high-dose MPS exhibited significant bone mineral loss and only a small amount of trabecular bone structure remains, comparing with the femur of normal rats. However, the number of trabeculae in the distal femur was partially improved after co-treated with PL, and significantly enhanced in exosome groups. Particularly, the modification of ALN amplified the efficacy of PL-derived exosomes, which were closed to the normal structure of heathy control (Fig. 6A). As for the quantitative analysis of BMD and trabecular morphometric parameters, decreased BMD, BV/TV, Tb. N and Tb. Th, as well as the increased Tb.Sp parameters were found in MPS groups, suggesting that overuse of MPS caused severe bone mass loss and destruction of trabeculae. While these parameters were reversed after native PL treatment, but there was no statistical significance except for BV/TV. Both two exosomes treatment groups displayed significant protective effect in the term of BMD, BV/TV, Tb. N and Tb.Sp. But in terms of the thickness of trabeculae, only PL-exo-ALN treatment could reverse MPS-mediated trabecular thinning.

According to the histology of undecalcified specimens, the dynamic bone formation and mineralization in the femoral bone was monitored by fluorochrome labeling with Alizarin red S and calcein, in the 3rd and 6th weeks. As shown in Fig. 6C, the strongest fluorochrome labeling with alizarin red S (red) and calcein (green) and the most trabeculae structure were found in the control group. What is more, the spacing of the two fluorescent lines was the largest in the control group, indicating substantial bone formation at weeks 3 to 6. Nevertheless, being treated with MPS decreased new bone formation and impaired bone homeostasis as evidenced by much weaker or even dissolved fluorochrome labeling in the trabeculae. Unfortunately, this phenomenon can hardly be altered by native PL treatment. By contrast, a much broader area of trabeculae was stained by fluorochrome labeling in the PL-derived exosomes group. Especially in PL-exo-ALN groups, the separation between two fluorescent lines was clearer and rarely overlapped, indicating osteogenesis restoration on GIOP after bone-targeted delivery of PL-exo (Fig. 6C). Further, the Von Kossa staining was applied to determine the mineralized bone area. As is presented in Fig. 6D, a significant decrease of mineralized bones was shown in MPS groups. On the contrary, the mineralized bone area of the distal femur of GIOP rats increased sequentially after native PL, PL-exo, and PL-exo-ALN treatments.

**Fig. 7:**
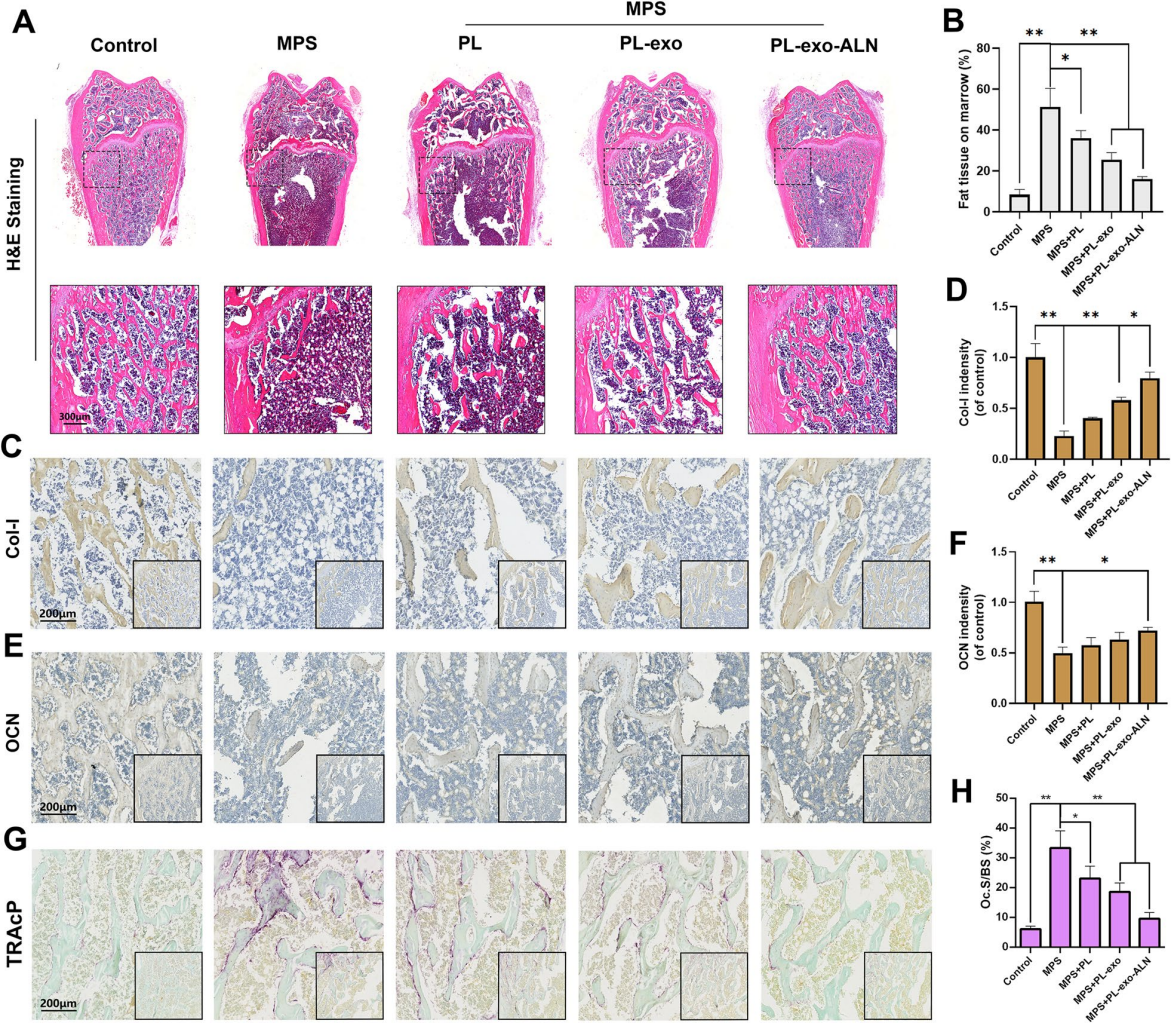
Effects of PL, PL-exo and PL-exo-ALN on MPS-induced fatty infiltration, down-regulation of osteogenic markers. **A** H&E staining of the distal femurs (scale bar: 300 μm). **B** Quantification of fat tissue on bone marrow. **C**–**F** Images and semi-quantifications for IHC staining of Col-I and OCN (scale bar: 200 μm). **G** TRAcP staining of the distal femurs (scale bar: 200 μm). **H** Quantitation of Oc.S/BS. All results are presented as the means ± SDs, ^*^P < 0.05, ^**^P < 0.01

For the histology of decalcified bone tissue, HE staining by paraffin section further confirmed the protection of PL, PL-exo and PL-exo-ALN on MPS induced bone loss, which was in keeping with the above undecalcified histology and micro CT analysis (Fig. 7A). By magnifying the HE images, plenty of fatty vacuoles infiltrated in the bone marrow of MPS-treated rats, whereas this condition was attenuated by PL, PL-exo and PL-exo-ALN in order (Fig. 7A–B). Moreover, the expression of Col-I and OCN, the crucial osteogenic proteins, was detected through IHC staining, helping to the deposition of mineralized bones. Consistent with the results of HE staining, the intensity of Col-I and OCN were both decreased significantly in MPS treated femur, but their expressions were increased in PL, PL-exo and PL-exo-ALN groups in turn (Fig. 7C–F). Based on the TRAcP staining in Fig. 7G–H, MPS-mediated osteoclast formation and activation was sequentially decreased after PL, PL-exo, and PL-exo-ALN treatments. Despite the lack of ALN molecular modification, the protection of PL and PL-exo partly benefits from the coordinated regulation of osteoblasts and osteoclasts by GFs. Additionally, we isolated the primary bone marrow monocytes (BMMs) and processed RANKL to induce osteoclasts formation in vitro. Based on the TRAcP staining visualization, we found not only the cell size but also the number of TRAcP-positive multinucleated osteoclasts was decreased after PL, PL-exo and PL-exo-ALN treatment especially PL-exo-ALN (Additional file 1: Figure S8A and B).

**Fig. 8:**
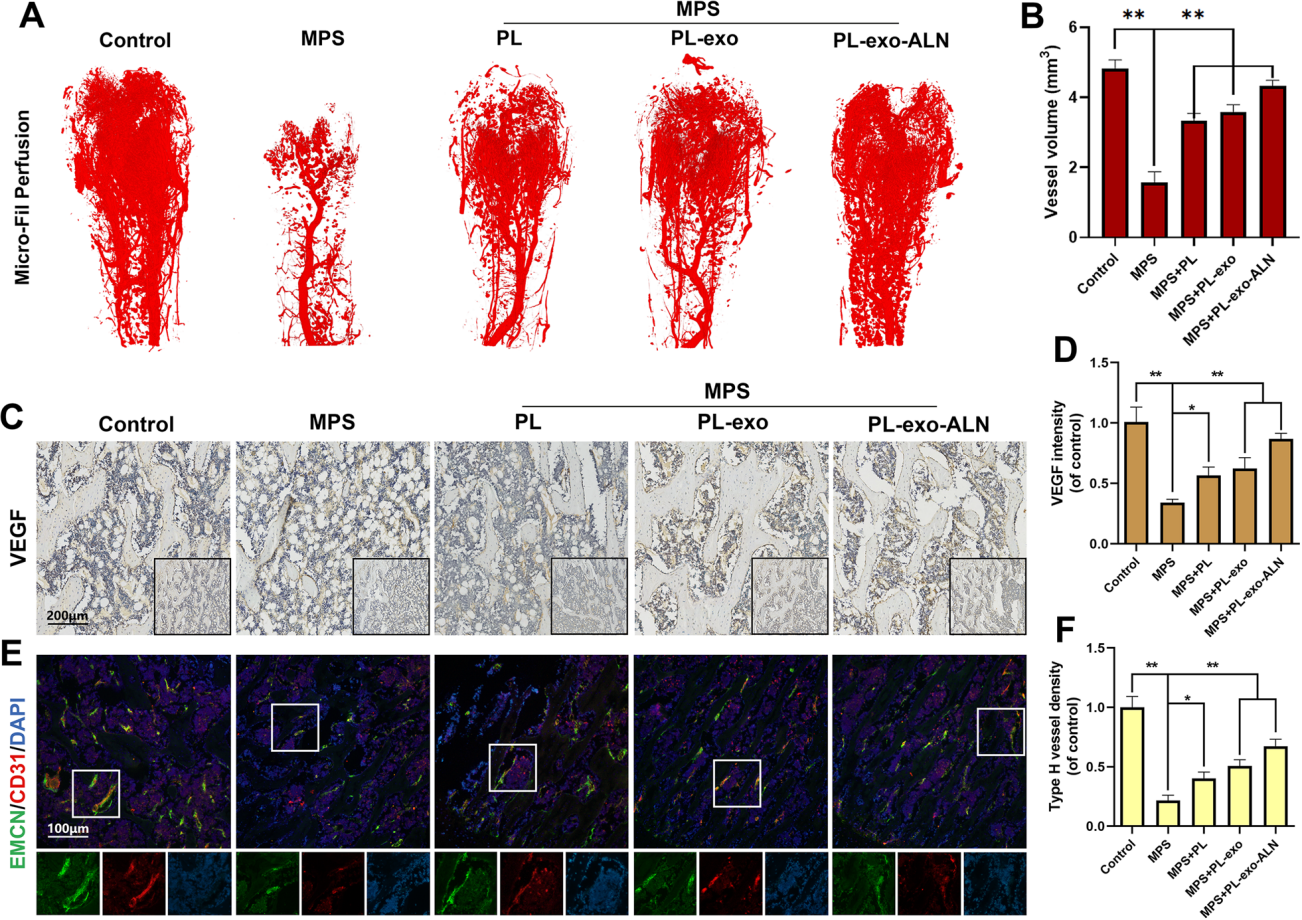
Effects of PL, PL-exo and PL-exo-ALN on MPS-induced vasculature destruction and decrease of angiogenic marker and H-type vessels. **A**–**B** Vessel perfusion and related quantification of five groups as indicated. **C**–**D** Images and semi-quantifications for IHC staining of VEGF. **E**–**F** Immunofluorescence staining of EMCN (green), CD31 (red) and nucleus (blue) (scale bar: 100 μm). All results are presented as the means ± SDs, ^*^P < 0.05, ^**^P < 0.01

As a highly vascularized tissue, osteogenesis requires the coordination of endothelial cell-derived molecules and the renewal of bone vasculature, but these processes are suppressed in GIOP. Our cellular experiments confirmed that PL and PL-derived exosomes not only directly accelerate angiogenesis, but also facilitate the osteogenic-angiogenic coupling by activating the FAK signal of BMSCs. To validate the angiogenesis effect of PL and PL-derived exosomes in vivo, rat femurs were perfused with Micro-Fil and scanned by Micro-CT after decalcification to observe the distribution of intra-osseous vessels. By 3D vascular reconstruction and quantitative analysis (Fig. 8A and B), PL, PL-exo and PL-exo-ALN treatment could restore the vascular damage in the distal femur caused by excessive MPS (PL < PL-exo < PL-exo-ALN), which was consistent with our IHC staining of VEGF (Fig. 8C and D). Besides, IF co-staining of endomucin (EMCN) and platelet endothelial cell adhesion molecule-1 (CD31) marked the type H vessels in the femur. Compared with the MPS group, the number of H-type vessels in the PL or PL-exosomes treated group increased, especially after PL-exo-ALN injection (Fig. 8E and F).

Furthermore, the biological safety of nanoparticles is of great concern [36]. Therefore, two months after MPS injection, the principal organs of each group of rats including the heart, liver, spleen, lung and kidney were collected and observed by HE staining. Remarkably, none of the rats died until the end of the animal experiment. Compared with normal rat liver, MPS-injected rats all showed hepatocyte shedding and vacuolation, enlarged central veins, and hepatic sinusoid disorders (Additional file 1: Fig. S5), which is due to the hepatotoxicity of high-dose MPS [37]. Our IVIS results indicated that systemic application of PL-exo and PL-exo-ALN partially accumulates in the liver. However, they are not hepatotoxic but did not significantly ameliorate MPS-mediated liver damage (Additional file 1: Figs. S5 and S6C). Except for the liver, no obvious signs of cell death or inflammatory cell infiltration were seen in other tissue sections. These data suggested that the PL, PL-exo and PL-exo-ALN has good systemic biocompatibility.

## Discussion

During past decades, owing to its potential for prompt bone regeneration, the application of GFs in orthopedics has drawn extensive attention [38, 39]. However, restricted by the issues of biosafety, productivity and cost-effectiveness, so far only a relatively small subset has achieved commercial transformation in orthopedics [40]. Meanwhile, despite considerable efforts to develop suitable carriers for GFs, most studies focus mainly on the local delivery of fracture but not on systemic administration for the whole skeleton [41, 42]. The primary concerns are the short half-lives, untargeted distribution, and underlying neoplastic risks [20]. Thus, despite the hype about the efficacy of GFs, solutions to the low-cost sources and delivery vehicles of GFs are still being actively debated.

PL as the second generation of PRP, is a cost-effective substitute of commercial recombined GFs, presenting broader application prospects [43]. In addition to replacing fetal bovine serum for the expansion of MSCs in vitro, PL accelerates bone repair in vivo by promoting osteogenesis and angiogenesis. As a plasma product, the primary problem to overcome is allogenic or even xenogenic immunogenicity. PL is purified from PRP by removal of cellular components so that it possesses lower immunogenicity and could be safely mixed with others. However, there is a large clinical demand for fresh platelet units, but their sources are limited from available blood donors. And the short storage time (~ 7 days half-life) of them exacerbates this contradiction. Fortunately, professor Sigurjonsson found that PLs extracted from expired platelet units have no significant difference in the proliferation and differentiation effects of MSC compared with fresh platelet units, so expired platelets might represent an alternative source of PL [44]. The underlying mechanism of PRP and PL was previously thought to be the release of GFs during platelet degranulation. Actually, even without a nucleus, platelets can secrete extracellular vesicles (microvesicles and exosomes) like cells after activation to participate in blood coagulation, inflammation, immunity and tissue regeneration, etc. [45]. The extracellular vesicles (EV) released by platelets account for approximately 25% of the total circulating plasma EV, under physiological conditions [46]. Interestingly, these exosomes can survive stably in the lysate after platelet rupture and are conveniently obtained by ultracentrifugation, which internally enriched GFs are considered to be the effector of PL [19]. Notably, proteomic analysis of PL indicated that 74.7% of PL's protein belongs to exosomal components, which is beneficial for effective intercellular communication of bioactive molecules [11]. Our cellular experiments suggested that PL-derived exosomes maintained the osteogenic differentiation of MSC and the vascularization of EPC more efficiently than PL. And the osteogenesis is similar to the conclusion of Professor Torreggiani [19]. We believe that PL-derived exosomes are expected to be the next generation alternative to PL based on this evidence.

The discovery of exosomes revealed a whole new way in which cells communicate with their neighbors. Indeed, exosomes are also recognized as an ideal nanocarrier for drug and gene transport due to their immune privilege, large loading capacity and cargo protection. And extraordinary surface molecular such as integrins makes the exosomes have inherent tissue and cell targeting capabilities, which only exist in certain types of exosomes like metastases [47]. Of course, the more specialized targeting ability of exosomes could be achieved through genetic engineering and chemical modification of surface structures [26, 48]. In present case, however, exosomes were directly extracted from PL without any cell cultural process, thus genetic engineering is not a proper method to choose. For chemical modification, the insertion of amphipathic molecules into the lipid bilayer of exosomes is one of the representative strategies for bone targeting [26]. And the PEGylation in nanoparticles was reported to help escaping from the reticuloendothelial system to prolong the blood circulation time, thereby might, to a certain degree, improve the circulated stability of GFs in PL-exo [49]. According to these, we immobilized DSPE-PEG-ALN on exosomes to successfully fabricate bone-targeting exosomes. Although oligopeptides like hexapeptide and aspartic acid octapeptide were also reported to have the bone-targeted ability [27, 28], ALN was finally chosen by considering the biosafety and cost-effectiveness issues, since it was FDA-approved and has been industrialized. What’s more, ALN alone also has potent pro-osteogenic abilities [29], thus incorporating PL-exo with ALN modification could synergistically facilitate bone formation, and this delivery system could appropriately resolve the restrictions of GFs’ systemic administration, including in vivo stability and targeted distributions.

Bone is a highly vascularized tissue, and its internal microvasculature provide niche signals for endothelial cells and peritubular cells in the bone microenvironment, which is the physiological basis of bone-vessel coupling. Except for inhibiting osteogenesis and activating osteoclasts, long-term exposure of GCs also damages H-type endothelial cells and hinders intraosseous angiogenesis, resulting in bone loss and even ischemic osteonecrosis [5, 50]. These pathological events frequently involve the dysregulation of signaling pathways (such as PDGFRβ/PI3K/AKT, VEGFR/MAPK, Wnt/β-catenin, HIF-α and Smad signals) caused by decreased bone-anabolic and angiogenic cytokines (such as PDGF-BB, VEGF, IGF and TGF-β) [9, 10]. In this study, the design of PL-exo-ALN is properly conformed to the pathophysiology of GIOP. Exosomes carried GFs could exogenously supplement GCs-induced deficiency of GFs and such a specified bone site infusion strategy of GFs not only directly rescued the GCs-induced inhibition of osteogenesis and angiogenesis, but also enhancing their coupling. This coupling effect was verified by upregulating the secretion of PDGF-BB and VEGF as well as BMP-2 and OPG by PL-derived exosomes, in BMSCs and EPCs, respectively. These results were partially consistent with E. Cenni et al. and Z. Hu et al. reported. Their studies found that PRP treatment could promote VEGF production in BMSCs, and BMP-2 and OPG production in endothelial cells [51, 52]. Although the PDGF-BB and VEGF were elevated in PL-exo treated BMSCs, which could promote the phosphorylation of FAK, thereby enhance the migration and vessel formation of EPCs. Accordingly, PL-exo drove the production of BMP-2 and OPG in EPCs, which facilitates the osteogenic differentiation of BMSCs to a certain degree. Especially, the OPG is a well-proven modulator in the crosstalk between vessels and bone [33]. Hence, our study demonstrated the predictable effects of PL-exo on regulation of osteogenesis and angiogenesis, but the molecular biological mechanism remains to be studied.

Osteoporosis caused by high-doses and long-term use of GCs is a complex process involving multiple cells. As for osteogenesis, excessive GCs promote the preferential differentiation of BMSCs into adipocytes rather than osteoblasts and mediate osteoblast apoptosis, thus decreasing the number of osteoblasts [5]. Exactly, physiological concentration of GCs is required for maintaining osteoblast viability and osteogenic differentiation. However, excessive GCs exposure mediates the up-regulation of Wnt antagonistic proteins including DKK1, Sost, sFRP-1and blocks the phosphorylation of AKT and GSK-3β, thereby degrading β-catenin and inactivating Wnt/β-catenin signaling [53]. PL and PL-exo, as a cluster of IGF, HGF and other GFs, form the complexes with growth factor receptors to activate the upstream signaling of PI3K/AKT and ERK1/2, which in turn stabilize β-catenin/TCF-dependent transcription by phosphorylating GSK-3β [23, 54, 55]. Additionally, various signal proteins involved in regulating adipo-osteogenic differentiation of BMSC. GCs upregulates adipocyte‐specific transcription factors such as PPARγ and C/EBPα, but downregulates Runx2 and TAZ, the crucial osteogenic transcription factor [56]. TGF-β, BMP-2 and FGFs contained in PL and PL-exo promote preferential osteogenic differentiation of BMSCs by coordinating Wnt/β-catenin, TAK1-MKK-MAPK, Notch, TGF-β/Smad and BMP/Smad signals [57–59]. Accumulating evidence shows that PL and PRP possess considerable anti-apoptotic and pro-proliferation effects. Wang et al. found that the addition of PL suppresses GCs-induced cell cycle arrest via G1/S transition and protects Mg63 cells against pro-apoptotic effects of GCs by increasing autophagy level [60]. Meanwhile, the osteoblasts death caused by GCs is partly mediated by endoplasmic reticulum stress, while PRP-exo activates the AKT/Bad/Bcl-2 axis to counteract the pro-apoptotic action triggered by eIF2α/ATF4/CHOP signal [23]. Based on the above researches, we speculated that the pro-differentiate, pro-proliferative and anti-apoptotic effects of PL or PL-exo are the core to promoting osteogenesis undergo GIOP.

Unlike cartilage, abundant microvascular network endows bone tissue with potent regenerative capacity. Arteries enter the long bones and differentiate into specific type L vessels (distributed in the medullary cavity to form vascular sinuses) and type H vessels (located in the endosteum and metaphysis); the latter provides niche signals for perivascular cells, enhancing osteogenic-angiogenic coupling [61]. The osteoblasts and preosteoclasts around H type vessels secrete Slit3, PDGF-BB, VEGF, angiogenin and so on to coordinate intraosseous angiogenesis. GCs application prevents intraosseous angiogenesis by decreasing the secretion of pro-angiogenic proteins and mediating endothelial cell apoptosis, clinically manifested as osteonecrosis [5]. PL is widely used in pre-clinical trials for wound repair and tissue regeneration, benefiting from abundant pro-angiogenic factors. Tang’s laboratory results showed that PL activates PI3K/AKT and MAPK/ERK1/2 signals to trigger mTOR/Hif-1α/VEGF-A axis, promoting endothelial cell proliferation and tube formation and migration [11]. And Professor Tao’s wound model proved that PRP-exo activates ROCK-mediated YAP nuclear transcription as a prerequisite for fibroblast migration and proliferation [21]. More importantly, except for GFs, PL and PL-exo are also effective carriers of microRNA, which is a significant cluster regulating bone homeostasis [62]. miRNA-27a was down-regulated in GCs-stimulated BMSC, whereas supplementing miRNA-27a promoted BMSC osteogenic differentiation by inhibiting PPARγ and GREM1 expression [63]. Apart from pro-osteogenic and anti-adipogenic ability, miRNA-20a directly interacts with the 3´-UTR of TNFSF15 to stimulate angiogenesis [64]. Similarly, miRNA-24 ameliorates cellular proliferation, apoptosis and mineralization of MC3T3-E1 cells infected with S. aureus [65]. Based on RNA sequencing, PL is rich in the above three microRNAs, which might further explain the protective effect of PL and PL-derived exosomes [66].

GCs therapy induced bone loss is mainly affecting the cancellous bone architecture, and its incidence and severity depend on the dose. In the current in vivo model, a relatively high dose of MPS was applied to properly mimic this severe bone destructive condition [65]. All MPS-exposed rats suffered from a dramatic bone loss of trabeculae and damaged vascularity in the distal femur. However, within the bone-targeted PL-exo administration, a significant improvement of bone mass and restoration of vasculature were found in PL-exo-ALN. At the same time, such a steady and targeted GFs enriched delivery strategy exert no side effects on other organs during all periods of experiments, which further demonstrated that this bone-targeted engineering PL-exo owns superior biosafety and has promising potential for GIOP treatment. Exactly, among the advantages of cost-effectiveness, low immunogenicity, easy separation, modification of exosomal membrane, PL-exo has broader application prospects than PL. Previously, PRP and its derivatives are widely used in clinical practice, but their burst-release effects, rapid degradation and unfavorable local retention have continued to confound physicians. As the ideal nanocarrier of GFs, exosomes, the double-layer lipid membrane effectively protects GFs from degrading enzymes or chemicals to achieve the sustained-release and stability of GF. Inserting targeted responsive molecules into lipid membranes achieves targeted delivery and controlled-release of GFs, which enables systemic injection of PL-exo without the risk of thrombosis and tumorigenesis. The chemcial modification of exosome membrane extremely expands the application fields of PL-exo, such as systemic osteopathia, spinal cord injury, etc.

## Conclusion

In brief, since PL is a cost-effective alternative to commercial recombined GFs, we isolated exosomes from PL and modified them with ALN molecules to achieve GFs enrichment and bone-targeted delivery. Constructed PL-exo-ALN showed a better HAp affinity in vitro and concentrated distribution in bone marrow in vivo, compared with PL-exo. During the GCs exposure, PL, PL-exo and PL-exo-ALN all contributed to the osteogenic differentiation of BMSCs and the migration and vascularization of EPCs, especially PL-derived exosomes. Furthermore, the potential crosstalk among BMSCs and EPCs was improved after treatment with PL, PL-exo and PL-exo-ALN. Meanwhile, intravenous administration of PL-exo-ALN could restore the severe bone mass loss and repair the disrupted blood supply, especially H-type vascular expression, in rats suffering from excessive MPS. Our works systemically evaluated the bone-targeting ability of PL-exo-ALN and its therapeutic potential for GIOP. Due to the modifiability of exosomes and the effective enrichment of GFs, we believe that PL-derived exosomes are expected to become a substitute for next-generation PL.

## Methods

### Synthesis and Characterization of the DSPE-PEG-ALN Conjugate

ALN was purchased from Aladdin (Shanghai, China). DSPE-PEG-NHS was ordered from Ruixi Bio-Tech Co, Ltd (Xi'an, China). The conjugating procedure was according to previous publications with slight modification [67]. Briefly, DSPE-PEG-NHS and ALN (at a 1:5 M ratio) were dissolved in DMSO, and then the pH of the solution was adjusted to 8.2 by using triethylamine. The resulting mixture was gently agitated at room temperature for 24 h, followed by being transferred to a dialysis bag (molecular weight cutoff = 3.5 kDa) and dialyzed against deionized water for 72 h to remove the unconjugated ALN. The obtained product (DSPE-PEG-ALN) was freeze-dried and stored at  − 20 ℃ until required. The Fourier transform infrared spectroscopy (FTIR, BrukerOptic Gmbh, Germany), and ^1^H-nuclear magnetic resonance (H-NMR, Bruker 400 M, USA) spectra were used to investigate the changes in chemical bonding alteration from the initial reagents to the final products.

### Extraction and ALN modification of PL-exosomes

Human platelet lysates (PL) were purchased from StemEry Hematopoietic Tech Co, Ltd (Fuzhou, China). Protocol for exosome isolation was based on a previously described method by Torreggiani et al. with mild emendation [19]. In brief, the PL was diluted five times using PBS firstly, followed by serial low-speed centrifugation (300×*g* for 10 min, 2000×*g* for 10 min and 10,000×*g* for 60 min) in 4 °C to remove cell debris. Then, the supernatant was collected and filtered through a 0.22 µm sterilized filter (Merck-Millipore, Darmstadt, Germany). The filtrate was pelleted by ultracentrifugation (Beckman ultracentrifuge, Beckman Coulter, USA) at 100,000×*g* for 70 min. The sediment was washed and resuspended in a large volume of PBS, and ultra-centrifuged again at the same high speed for 70 min. The final precipitated PL-exos was carefully resuspended in PBS, and the protein content of exosomes and native PL were quantified by using BCA assay according to the manufactural instructions. Finally, they were stored at  − 80 °C for subsequent experiments.

Modification of exosomes’ membrane with phospholipid polymer referred to previous studies [68]. The purified DSPE-PEG-ALN was dissolved in HEPES (4-(2-Hydroxyethyl)-1-piperazineethanesulfonic acid) buffer for 15 min at 60 °C to form micelles. Then, the obtained suspension was mixed with PL-exo solution at a 1:1 mass ratio for 2 h at 40 °C. After cooling to room temperature, exosomes were immediately purified by size-exclusion chromatography (Exo-spin™, XP Biomed Ltd. Shanghai, China) to get ALN-modified exosomes (PL-exo-ALN). The morphology, size distribution and zeta potential of exosomes were identified by using TEM (Hitachi, Tokyo, Japan), dynamic light scattering (DLS) (Beckman delsa, Brea, USA) and Zetasizer (Malvern, UK).

### In vitro hydroxyapatite binding assay

Quartz crystal microbalance with dissipation (QCM-D, Q-sense E1, Biolin, Sweden) incorporated with hydroxyapatite (HAp) coated sensor (QSX 327, Biolin, Sweden) were first used to determine the in vitro bone-targeting ability of exosomes. Briefly, the sensors were cleaned for 30 min in 99% ethanol, rinsed with deionized water, and exposed under UV for 10 min. They were then mounted in the flow cells, and PBS was injected for 5 min (100 µL/min) to allow the signal to be stabilized. Subsequently, a 10 µg/mL solution of PL-exo or PL-exo-ALN in PBS was injected at the flow speed of 20 µL/min for 150 s to enable the film surface to be in contact with the exosome solution. Finally, the surfaces were rinsed with PBS for 5 min to exclude unstable binding of exosomes and to ensure signal stability. The third overtone frequency of the sensor was used to assess the exosomes’ disposition on the HAp surface. Changes in the resonance frequency (Δf) and dissipation (ΔD) were recorded.

Another HAp binding method was also used to mimic the binding affinity of Aln functionalized exosomes to bone surface in vitro by using DiD (a kind of lipophilic fluorochrome) labeled exosomes. Firstly, the PL-exo and PL-exo-ALN solutions were incubated with 3 μM DiD for 1 h at room temperature to obtain fluorescent exosomes. Then, they were incubated with HAp (10 mg/mL) suspensions with gently shaken for 5 h at 25 °C, followed by centrifugation at 4000 rpm for 10 min to spin down HAp and exosomes bound to them, the gross images were captured by IVIS imaging (Lumina Series III, PerkinElmer, USA). And the fluorescence intensities of the supernatants were measured using fluorimetry (Edinburgh Instruments FS920, Ex 474 nm, Em 533 nm). The decrease in the intensity relative to the initial intensity suggests the degree of exosome bound to Hap.

### Cell culture and treatment

Rat bone marrow mesenchymal stem cells (BMSCs) and endothelial progenitor cells (EPCs) were harvested from the marrow of healthy 2‐week‐old Sprague‐Dawley rats according to our previous studies [69, 70]. BMSCs were cultured with α‐MEM (HyClone, Shanghai, China) supplemented with 1% penicillin/streptomycin (Gibco, Shanghai, China) and 10% fetal bovine serum (FBS, Gibco, Shanghai, China). EPCs were cultured in endothelial cell growth media (EGM-2) supplemented with EGMTM-2 MV SingleQuots^™^ (Lonza, Basel, Switzerland). Both cells were seeded in 5% CO_2_ at 37 ℃. All the procedures were approved by the Institutional Ethics Review Committee of Wenzhou Medical University. A high dose of dexamethasone (Dex) was applied to mimic the GC-induced detrimental effects on cells. The concentration of PL (50 µg/mL), exosomes (50 µg/mL) and Dex (10 μM) were according to previous publications [23].

To evaluate the effect of PL, PL-exo and PL-exo-ALN on osteoclasts, bone marrow monocytes (BMMs) were harvested from the marrow of healthy 6-week-old C57BL/6 mice according to previous study [71]. BMMs were cultured with α‐MEM (HyClone, Shanghai, China) supplemented with 1% penicillin/streptomycin (Gibco, Shanghai, China), 10% fetal bovine serum (FBS, Gibco, Shanghai, China) and 50 ng/mL M-CSF (Peprotech, NJ, USA). As for the oteoclast differentiation, the BMMs (6 × 10^3^ cells/well) were seeded on 96-well plates and incubated with 50 ng/mL RANKL (Peprotech, NJ, USA) after 24 h. PL (50 µg/mL) and exosomes (50 µg/mL) were added to the related medium in the experimental groups, while the control group was cultured in α‐MEM complete medium containing M-CSF without any addition. The medium was replaced every two days until osteoclasts formed on the sixth day. Cells were then fixed and visualizated by tartrate-resistant acid phosphatase (TRAcP) staining. TRAcP-positive multinucleated cells that had more than three nuclei were counted as osteoclasts. The number of osteoclasts per well was used to evaluate the effect on the inhibition of osteoclastogenesis.

### In vitro and in vivo distribution of exosomes

To evaluate the internalization of exosomes in vitro, the PL-exo and PL-exo-ALN were labeled with the DiD firstly as mentioned above. Then, BMSCs were exposed to the labeled exosomes for 12 h. After that, the cells were fixed with 4% paraformaldehyde (PFA) for 30 min at room temperature, followed by permeabilized in 0.1% Triton-X 100 in PBS for 5 min, and stained cytoskeleton and nucleus with FITC labeled phalloidin (Yeasan, Shanghai, China) and 4′,6-diamidino-2- phenylindole (DAPI, Beyotime Biotechnology, China) respectively. The images were obtained using an invert fluorescence microscope (Olympus, Japan).

The bone targeting effect of PL-exo-ALN was evaluated in vivo by assessing the biodistribution of the intravenous delivery of DiD labeled exosomes. Six female Sprague–Dawley rats (200–250 g, 8-week-old) were obtained from the Animal Center of the Chinese Academy of Science (Shanghai, China). The rats were divided into two groups: PL-exo and PL-exo-ALN (n = 3 in each group). After anesthesia with sodium pentobarbital, the rats were received 100 μg exosomes (dissolved in 200 μL of PBS) via tail vein injection. Six hours later, the rats were sacrificed and the femur along with major organs (femur and heart, liver, spleen, lung, kidney) were collected for IVIS imaging (Lumina Series III, PerkinElmer, USA).

### Cell viability assay

Cell counting kit-8 (CCK-8, Dojindo, Japan) was performed to assess the cell viability. A total of 5000 BMSCs per well were seeded into 96-well plates. One group without treatments served as the control. The remaining groups were treated with Dex (10 μM). During the Dex exposure, PL, PL-exo and PL-exo-ALN (50 µg/mL) immediately added to each group. After culturing for three days, in each well, 90-µL medium and 10-µL CCK-8 solution were topped up and incubated at 37 °C for one hour. A microplate reader (Thermo Fisher Scientific, MA, USA) was used to determine the viability of the cells at absorbance 450-nm.

### Osteogenic differentiation

The method for osteogenic differentiation was depending on a previous study [69]. Briefly, the BMSCs (1 × 10^4^ cells/cm^2^) were seeded on 24-well plates and incubated with osteogenic induction medium (OM, containing 1 nM dexamethasone, 50 μM L-ascorbic acid-2-phosphate and 20 mM β-glycerophosphate), after reaching 80% frequency. Dex and exosomes were added to the related medium in the experimental groups, while the control group was cultured in OM without any addition. The medium was replaced every two days. Alkaline phosphatase (ALP) activity was measured after 5 days of culture by stained with BCIP/NBT ALP Color Development Kit (Beyotime, Shanghai, China) and quantified with an alkaline phosphatase assay kit (Beyotime, Shanghai, China) after lysed. The calcium deposits were evaluated by Alizarin red staining (Cyagen Biosciences, Guangzhou, China) after 14 days of culture, and the stained mineralized nodules were desorbed with 10% cetylpyridinium chloride (Sigma-Aldrich) and the OD value was measured at 570 nm for quantification.

### Western blotting

After treatment, total cell protein was extracted by using RIPA lysis buffer containing 1% phenylmethanesulfonyl fluoride (PMSF). The nuclear protein extraction was achieved by using the nuclear protein and cytoplasmic protein extraction kit from Thermo Fisher Scientific. The extracts were lysed on ice for 30 min, followed by treatment with ultrasound, and then centrifuged at 12,000 rpm for 20 min at 4 °C. After quantified by BCA kits, a total of 30 μg of the cellular protein was diluted with ddH_2_O and loading buffer (Beyotime, China) to establish a 20 ul sample system. But for exosome, only 10 μg exosomal protein is contained in 20 ul sample system. The samples were separated with sodium dodecyl sulfate–polyacrylamide gel electrophoresis (SDS-PAGE) and then blotted onto a polyvinylidene difluoride membrane (Bio-Rad, Hercules, CA, USA). Blocking was carried out for 2 h using 5% BSA, and subsequent incubation of the membranes together with a primary antibody against Col-I (1:1000, abcam, USA), Runx-2 (1:1000, Bioworld, USA), OCN (1:500, santa, USA), p-PDGFRβ1(1:1000, Cell Signaling Technology, USA), PDGFRβ1(1:1000, Cell Signaling Technology, USA), p-FAK(1:1000, abcam, USA), FAK(1:1000, abcam, USA), PDGF-BB(1:1000, LifeSpan BioSciences, USA), VEGF-A(1:1000, abcam, USA), BMP-2(1:1000, abcam, USA), OPG(1:1000, abcam, USA) and GAPDH (1:10,000, abcam, USA) and exosomal markers CD9 (1:1000; Abcam, Cambridge, UK), CD81 (1:1000; Abcam), TSG101 (1:1000; Santa Cruz, Dallas, USA), the platelet marker CD41 (1:1000; Santa Cruz) and cytoplasm marker Calnexin (1:1000; Santa Cruz) at 4℃ overnight. The next day, the membrane was incubated for 2 h with the respective secondary antibodies at room temperature. After being washed three times using TBST, the blots were then developed using the Bio-rad ChemiDoc MP (Bio-Rad, USA). Finally, band intensity was evaluated using image lab 3.0 software (Bio-Rad, USA). The data were normalized and presented as the fold change of the protein level of the treatment group compared to the control group.

### Immunofluorescence

The cells were plated on crystal 6-well slice at the density of 5 × 10^5^/ml in the culture plates, and treated with Dex and different groups of exosomes for 24 h. The samples were washed thrice in PBS, fixed in 4% paraformaldehyde, and permeabilized for 15 min with 0.1% TritonX-100 in PBS. The 5% bovine serum albumin was used to block cells at 37 °C for 1 h, followed by washing with PBS, and culturing with primary antibodies against Col-I (1:200, abcam, USA), or overnight at 4 °C. The TRITC Phalloidin, Alexa Fluor^®^488-labeled secondary antibodies (1:400, abcam, USA) and DAPI solution (1:10) were added in sequence for 20 min, 1 h and 5 min at room temperature respectively, followed by 3 times rinsing in PBS. The images were captured by an inverted fluorescence microscope (Olympus, Japan). As for histofluorescence, the sections were deparaffinized and hydrated and incubated with a mixture of EMCN (1:200, Invitrogen, USA) and CD31(1:200, Invitrogen, USA). After washed with PBS, sections were incubated with secondary antibodies: Alexa Fluor 488-conjugated and Alexa Fluor 594-conjugated (1:200, Abcam) at room temperature for 30 min. The nuclei were stained with DAPI solution. The images were captured by an inverted fluorescence microscope (Olympus, Japan).

### In vitro tube formation

The tubule formation ability of EPCs after treatments was tested by transferring the cells seeding on Matrigel™ (BD Bioscience). In brief, a 96-well plate was coated with 50 μL of cold Matrigel per well and gelatinized at 37 °C for 30 min. Then, EPCs suspension of 2.0 × 10^4^ cells/mL were digested and seeded on the matrix. After 6 h of incubation, the number of complete capillaries and nodes of each hole were calculated.

### Migration assay

To measure the migration potential of EPCs after different treatments, a transwell assay was used. The cells were seeded in the upper chambers of a 24-well transwell plate (Corning, USA) at a density of 5.0 × 10^4^ cells/mL. Serum-free mediums were added to the lower chambers. And Dex and different groups of PL or PL-exosomes were added to serum-free medium. After 24 h of incubation, the cells on the upper surface of the transwell membrane were gently wiped with a cotton swab, and cells on the lower surface were fixed with 4% paraformaldehyde and stained for 10 min with 0.5% crystal violet. Finally, 3 random lower surfaces of each filter were chosen and counted twice.

### Preparation of conditioned media from BMSCs and EPCs

BMSCs or EPCs were treated with PL, PL-exo, or PL-exo-ALN for 24 h or not without treatment, then the serum-containing conditioned media (CM) were collected. After centrifugation (2500 rpm for 10 min at 4 °C), we obtained the supernatants and stored them at -80 °C for downstream experiments. After seeding the BMSCs or EPCs, we changed to the corresponding CMs and added Dex (10 μM) for culture. As for the migration experiment, we also harvested different types of serum-free CM from BMSCs after 24 h of the above treatments. After inoculating EPCs in the upper chambers of a 24-well transwell plate, Dex (10 μM) and corresponding serum-free CMs were added to the lower chambers.

### Animal model

All procedures followed in our study for animal care and use complied with the Guides for the Care and Use of Laboratory Animals of the National Institutes of Health and was approved by the Animal Care and Use Committee of Wenzhou Medical University. A total forty male Sprague–Dawley rats (220–250 g, 6-week-old) were provided by the Animal Center of the Chinese Academy of Science (Shanghai, China). The rats were randomly divided into five groups: Control, MPS, MPS + PL, MPS + PL-exo, MPS + PL-exo-ALN (n = 8 in each group) and housed in standard temperature conditions with a 12-h light/dark cycle and regularly fed with food and water. The establishment of GIOP model was according to previous studies [72]. Rats in the MPS-related groups were received an intramuscular injection of methylprednisolone (MPS) (30 mg/kg/day in 0.9% NaCl solution) for 60 days, while a control group was injected daily with equal volume vehicle (saline). After 3 weeks injection of MPS, the treatment groups were intravenously administrated with PL, PL-exo, PL-exo-ALN (100 μg in PBS) once a week. In contrast, the MPS groups received equal volume PBS treatment only. The injection dose and frequency of PL and PL-exo are performed by referring to relevant literature [23, 73, 74].

To monitor dynamic bone formation and mineralization, five rats of each group were intraperitoneally injected with 30 mg/kg alizarin red S (Macklin, Shanghai, China) and 10 mg/kg calcein (Macklin, Shanghai, China) at week 3 and 6 during the experiment. At the end of the treatment period, these five rats were humanely sacrificed and femurs were collected for micro-CT scanning. Then, the right femurs were used for undecalcified histological examination, while the left femurs were decalcified in 10% EDTA for hematoxylin and eosin (H&E), tartrate‐resistant acid phosphatase (TRAcP) and immunohistological staining. On the other hand, the rest three rats in each group were anaesthetized and perfused with Microfil to assess intraosseous vessels. Then, the femurs were collected and decalcified with 10% EDTA for two months. After micro-CT scanning, femurs were paraffin-embedded for immunofluorescence staining. The investigators were not blinded to allocation during experiments and outcome assessment. Sample sizes were selected on the basis of previous experiments. No animals were excluded from analysis.

### Micro-CT scanning

Micro-CT scanning and corresponding analysis for femurs were performed using SkyScan1178 system and bundled software (Bruker MicroCT, Kontich, Belgium). The 14-micron voxel size of images were acquired with a set at 35 kV of energy and 220 mA of intensity. 2D and 3D reconstructions were performed using DataViewer and CTVox software respectively. We chose the trabecular bone at the metaphysis of the distal femur below the growth plate as a region of interest (ROI). The bone mineral density (BMD), trabecular bone volume fraction (BV/TV), trabecular number (Tb.N), trabecular thickness (Tb.Th) and trabecular separation (Tb.Sp) were calculated by using CTAn software.

### Histology

For undecalcified sections, after embedding in resin, specimens were sawn coronally into 100-μm thickness, fixed on plastic slide, and carefully grinded and polished into about 50-μm thickness. The immunofluorescent signals about dynamic bone formation were captured using a laser scanning confocal microscope (Leica Microsystems, Wetzlar, Germany). The later Von Kossa staining was performed by immersing slices into 5% silver nitrate for 3 h in 60 ℃ oven and cleaned with sodium thiosulfate solution. The images were recorded using microscope (Olympus, Japan).

For decalcified specimens, after being dehydrated using an alcohol gradient, cleared, and embedded in paraffin, the tissues were cut into 5-μm-thick sections. H&E and TRAcP were performed as described previously [75]. The osteoclast surface per bone surface (Oc.S/BS) were calculated using BIOQUANT OSTEO 2011 software according to a method proposed by Sawyer et al. [76]. For immunohistochemical (IHC) staining, sections were deparaffinized, antigen retrieved, blocked and incubated with primary antibodies of COL I (1:200, abcam, USA), OCN (1:100, santa, USA) and VEGF (1:100, abcam, USA) and relevant biotinylated secondary antibodies. Finally, sections were stained with DAB and counterstained with hematoxylin. The light images were captured using microscope (Olympus, Japan). Semi-quantitative analysis was carried out using Image-Pro Plus software version 6.0 (Media Cybernetics, Rockville, MD, USA).

### Micro-fil perfusion

Three rats of each group were applied for Micro-fil perfusion to assess intraosseous vessels, according to previous publications [23, 77]. After anesthesia, opened the thoracic and abdominal cavity to expose and dissect the abdominal aorta and vein and the proximal aorta. After ligating the proximal aorta and cutting off the abdominal vein, a needle was inserted into the abdominal aorta. And the vasculature was flushed with heparinized saline (0.9% normal saline containing 100 U/ml heparin sodium), 4% paraformaldehyde (PFA) was then injected for fixation. After the lower limbs were fixed and stiff, Micro-fil (MV-122, Carver, MA, USA) were injected into the abdominal aorta until a constant outflow was shown in the abdominal vein. About 25 ml Micro-fil was consumed per rat to ensure adequate filling of intraosseous vessels. The rats were stored at 4 °C overnight to ensure polymerization. Then, the bilateral femurs were removed, fixed and decalcified with 10% EDTA solution for 2 months. Finally, internal femoral vessels were imaged and analyzed by Micro-CT. The scanner was set at a resolution of 9 μm per pixel. The 3D images of the vasculature were reconstructed using CTVox software. The total vessel volume was calculated using CTAn software.

### ELISA assay

ELISA kits detecting the level of bFGF, PDGF-AB, VEGF, TGF-β1 and PDGF-BB were obtained from R&D systems. Assays were performed according to the manufacturer's instructions. Absorbance at 450 nm in the ELISA assays was detected on a microplate reader (Thermo Fisher Scientific, MA, USA).

### Statistical analysis

At least three independent replicates were performed. The data are presented as the mean ± standard deviation (SD). We used unpaired, two-tailed Student’s t-tests for comparisons between two groups and one-way analysis of variance (ANOVA) with Tukey’s multiple comparisons test for multiple comparisons. All statistical analyses were performed by Prism software version 8.0 (GraphPad, San Diego, CA, USA). A P value < 0.05 was considered statistically significant. Power analysis was performed by G*power software (Version 3.1).

## Supplementary Information

**Additional file 1**: **Figure S1. **FTIR spectra assay of ALN, DSPE-PEG-NHS and DSPE-PEG-ALN. **A** FTIR spectra result of ALN. **B** FTIR spectra result of DSPE-PEG-NHS. The intensity of peak at 1,733 cm^-1^, correspondent to C=O stretch of NHS groups. **C** FTIR spectra result of DSPE-PEG-ALN. The characteristic peak of NHS groups is strongly reduced in the DSPE-PEG-ALN, which is consistent with nucleophilic substitution of NHS for alendronate. **Figure S2.** ^1^H-NMR spectroscopy result of ALN. **A** Chemical structure of ALN. **B** ^1^H-NMR results of ALN in the range of 1.2ppm to 3.2 ppm. The characteristic signal at 1.88 and 2.93 ppm corresponding to CH_2_ protons. **Figure S3**. ^1^H-NMR spectroscopy result of DSPE-PEG-NHS. **A** Chemical structure of DSPE-PEG-NHS. **B** ^1^H-NMR results of DSPE-PEG-NHS in the range of 0 ppm to 10 ppm, evidencing the main peak of PEG chain (3.64 ppm). **C** ^1^H-NMR results of DSPE-PEG-NHS in the range of 0 ppm to 4.6 ppm, evidencing the carbon lateral chains (1.25 ppm), terminal methyl groups (0.80 ppm) and NHS (2.67 ppm, arrow). **Figure S4. **^1^H-NMR spectroscopy result of DSPE-PEG-ALN.**A** Chemical structure of DSPE-PEG-ALN. **B** ^1^H-NMR results of DSPE-PEG-ALN in the range of 0 ppm to 10 ppm, evidencing the main peak of PEG chain (3.64 ppm). **C**^1^H-NMR results of DSPE-PEG-ALN in the range of 0 ppm to 3.7 ppm showed a reduction of NHS group signal (2.67 ppm) and the appearance of ALN characteristic signal (1.98 and 3.13 ppm). **Figure S5.** H&E staining of heart, liver, spleen, lung and kidney in five groups. **Figure S6**. **A** Zeta potential value of PL, PL-exo and PL-exo-ALN. **B** The cell viability of BMSCs treated as above was detected by CCK-8. **C** H&E staining of liver in the above groups. **D** The osteogenesis- and angiogenesis-associated growth factors in PL and PL-exo was determined by ELISA. All results are presented as the means ± SDs, *P < 0.05, **P < 0.01.** Figure S7.** Effects of PL-exo on osteogenic and angiogenic differentiation of BMSCs and EPCs. **A**–**C** Early osteogenic differentiation was determined by ALP staining and ALP activity assays after 5 days of induction. Late osteogenic differentiation was determined by Alizarin Red staining and the calcium deposition was quantified by measuring the optical density, after 14 days of induction. **D**–**E** *In vitro* tube formation assay of EPCs treated as indicated (scale bar: 150 μm). **F**–**G** The expression levels of p-AKT, AKT, Col1 and β-catenin in BMSCs treated as indicated for 36 h. Exosomes (50 µg/mL) and MK-2206 (5μM, a selective inhibitor of AKT) were added to the related medium in the experimental groups, while the control group was cultured in osteogenic induction medium without any addition. **H**–**I** The expression levels of p-AKT, AKT, VEGF and Hif-α in EPCs treated as indicated for 24 h. Exosomes (50 µg/mL) and MK-2206 (5μM) were added to the related medium in the experimental groups, while the control group was cultured in EGM-2 complete medium without any addition. All results are presented as the means ± SDs, ^*^P < 0.05, ^**^P < 0.01.** Figure S8.** Effects of PL, PL-exo and PL-exo-ALN on RANKL-induced osteoclastogenesis in BMMs. **A** Representative images of TRAcP-stained osteoclasts after treated with PL, PL-exo and PL-exo-ALN. **B** The quantitative analysis of TRAcP-positive multinucleated cells (>3 nuclei) per well (96-well plate). All results are presented as the means ± SDs, **P < 0.01. **Table S1**. Post hoc power analyses of the different experiments (Unpaired t-tests). **Table S2.** Post hoc power analyses of the different experiments (One-way ANOVA).

## Data Availability

The datasets used and analysed during the current study are available from the corresponding author on reasonable request.

## Abbreviations

GCs: Glucocorticoids
GFs: Growth factors
PL: Platelet lysates
PL-exo: PL-derived exosomes
ALN: Alendronate
ALN: Conjugating with alendronate
DSPE-PEG-ALN: Grafted PEGylated phospholipid
PL-exo-ALN: ALN-modified PL-exo
BMSCs: Bone marrow mesenchymal stem cells
EPCs: Endothelial progenitor cells
GIOP: GCs induced osteoporosis
PDGF-BB: Platelet-derived growth factor type BB
VEGF: Vascular endothelial growth factor
IGF-1: Insulin-like growth factor-1
TGF-β: Transforming growth factor-β
bFGF: Basic fibroblasts growth factor
PRP: Platelet-rich plasma
PFA: Paraformaldehyde
ALP: Alkaline phosphatase
BMD: Bone mineral density
BV/TV: Trabecular bone volume fraction
Tb.N: Trabecular number
Tb.Th: Trabecular thickness
Tb.Sp: Trabecular separation
MPS: Methylprednisolone
