# Supplementary material for: Bone-targeting delivery of platelet lysate exosomes ameliorates glucocorticoid-induced osteoporosis by enhancing bone-vessel coupling
Source: J Nanobiotechnology. 2022 Oct 31;20:220. doi: 10.1186/s12951-022-01400-1 (PMC9620632; doi:10.1186/s12951-022-01400-1)
Supplement: Supplementary file 1 — Additional file 1: Figure S1. FTIR spectra assay of ALN, DSPE-PEG-NHS and DSPE-PEG-ALN. A FTIR spectra result of ALN. B FTIR spectra result of DSPE-PEG-NHS. The intensity of peak at 1,733 cm-1, correspondent to C=O stretch of NHS groups. C FTIR spectra result of DSPE-PEG-ALN. The characteristic peak of NHS groups is strongly reduced in the DSPE-PEG-ALN, which is consistent with nucleophilic substitution of NHS for alendronate. Figure S2. 1H-NMR spectroscopy result of ALN. A Chemical structure of ALN. B 1H-NMR results of ALN in the range of 1.2ppm to 3.2 ppm. The characteristic signal at 1.88 and 2.93 ppm corresponding to CH2 protons. Figure S3. 1H-NMR spectroscopy result of DSPE-PEG-NHS. A Chemical structure of DSPE-PEG-NHS. B 1H-NMR results of DSPE-PEG-NHS in the range of 0 ppm to 10 ppm, evidencing the main peak of PEG chain (3.64 ppm). C 1H-NMR results of DSPE-PEG-NHS in the range of 0 ppm to 4.6 ppm, evidencing the carbon lateral chains (1.25 ppm), terminal methyl groups (0.80 ppm) and NHS (2.67 ppm, arrow). Figure S4. 1H-NMR spectroscopy result of DSPE-PEG-ALN.A Chemical structure of DSPE-PEG-ALN. B 1H-NMR results of DSPE-PEG-ALN in the range of 0 ppm to 10 ppm, evidencing the main peak of PEG chain (3.64 ppm). C1H-NMR results of DSPE-PEG-ALN in the range of 0 ppm to 3.7 ppm showed a reduction of NHS group signal (2.67 ppm) and the appearance of ALN characteristic signal (1.98 and 3.13 ppm). Figure S5. H&E staining of heart, liver, spleen, lung and kidney in five groups. Figure S6. A Zeta potential value of PL, PL-exo and PL-exo-ALN. B The cell viability of BMSCs treated as above was detected by CCK-8. C H&E staining of liver in the above groups. D The osteogenesis- and angiogenesis-associated growth factors in PL and PL-exo was determined by ELISA. All results are presented as the means ± SDs, *P < 0.05, **P < 0.01. Figure S7. Effects of PL-exo on osteogenic and angiogenic differentiation of BMSCs and EPCs. A–C Early osteogenic differentiation was determined b [file 12951_2022_1400_MOESM1_ESM.docx]

**Bone-targeting delivery of platelet lysate exosomes ameliorates glucocorticoid-induced osteoporosis by enhancing bone-vessel coupling**

**Gang Zheng^1,2^,** **Hai-Wei Ma^1,2^, Guang-Heng Xiang^1,2^, Gao-Lu He^1,2^, Han-Chen Cai^1,2^, Zi-Han Dai^1,2^, Yan-Lin Chen^3^, Yan Lin^1,2^, Hua-Zi Xu****^1,2^, Wen-Fei Ni^1,2*^, Cong Xu^1,2*^, Hai-Xiao Liu^1,2*^, Xiang-Yang Wang^1,2*^**

1. Key Laboratory of Orthopaedics of Zhejiang Province, Department of Orthopaedics, The Second Affiliated Hospital and Yuying Children’s Hospital of Wenzhou Medical University, Wenzhou 325000, Zhejiang Province, China

2. The Second School of Medicine, Wenzhou Medical University, Wenzhou 325000, Zhejiang Province, China

3. Department of Orthopaedic Surgery, Lishui Central Hospital and Fifth Affiliated Hospital of Wenzhou Medical University, Lishui 323000, Zhejiang Province, China

*Wen-Fei Ni

Department of Orthopaedics, The Second Affiliated Hospital and Yuying Children’s Hospital of Wenzhou Medical University, Wenzhou 325000, Zhejiang Province, China

Email: wenfeini@yeah.net

*Cong Xu

Department of Orthopaedics, The Second Affiliated Hospital and Yuying Children’s Hospital of Wenzhou Medical University, Wenzhou 325000, Zhejiang Province, China

Email: derek1226@163.com

*Hai-Xiao Liu

Department of Orthopaedics, The Second Affiliated Hospital and Yuying Children’s Hospital of Wenzhou Medical University, Wenzhou 325000, Zhejiang Province, China

Email: spineliu@163.com

*Xiang-Yang Wang

Department of Orthopaedics, The Second Affiliated Hospital and Yuying Children’s Hospital of Wenzhou Medical University, Wenzhou 325000, Zhejiang Province, China

Email: xiangyangwang@wmu.edu.cn

Gang Zheng, Hai-Wei Ma, Guang-Heng Xiang contributed equally to this work.

**1. Additional file 1: Figures**


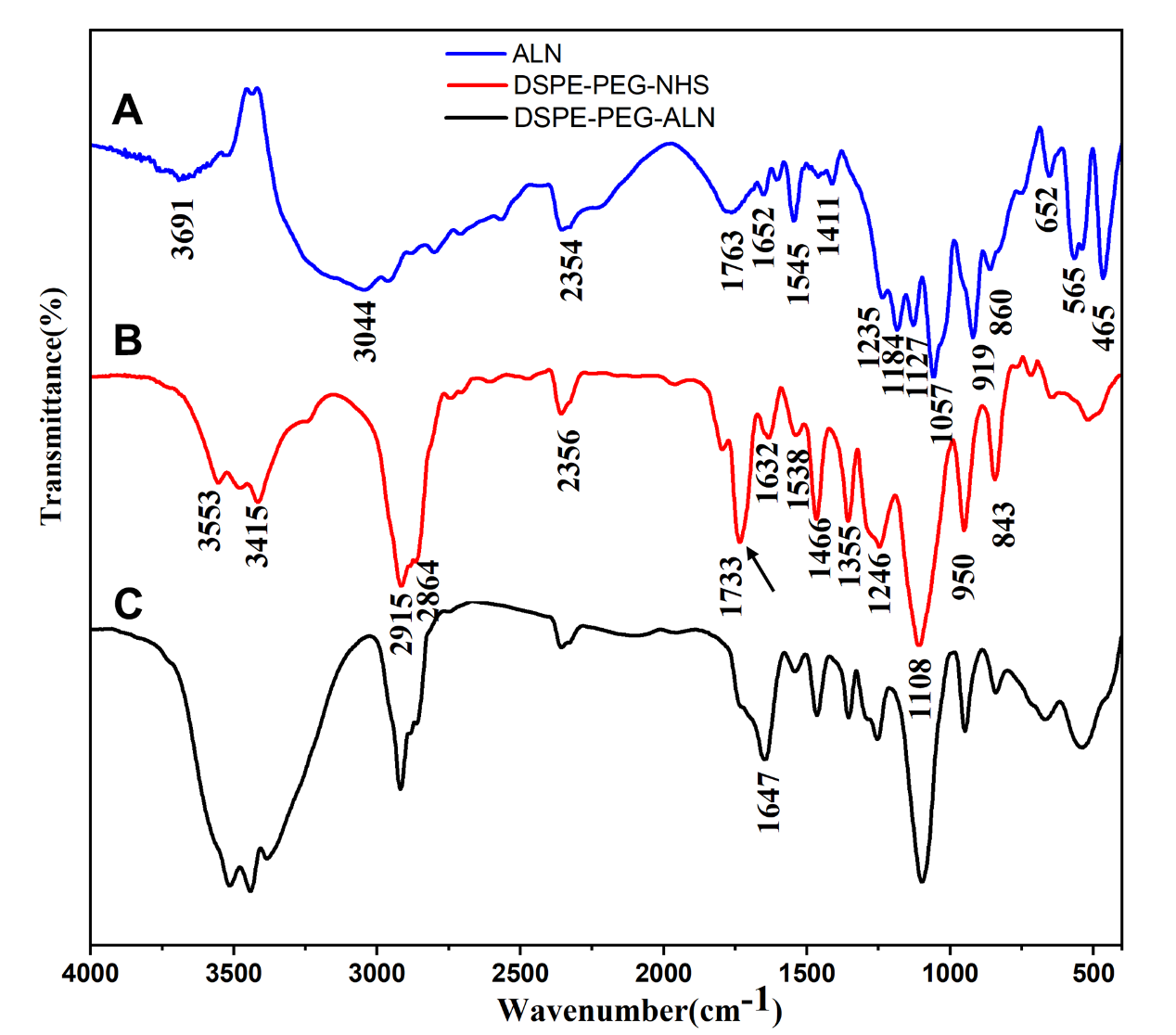
 **Additional file 1: Figure S1. FTIR spectra assay of ALN, DSPE-PEG-NHS and** **DSPE-PEG-ALN.**

(A). FTIR spectra result of ALN. (B). FTIR spectra result of DSPE-PEG-NHS. The intensity of peak at 1,733 cm^-1^, correspondent to C=O stretch of NHS groups. (C). FTIR spectra result of DSPE-PEG-ALN. The characteristic peak of NHS groups is strongly reduced in the DSPE-PEG-ALN, which is consistent with nucleophilic substitution of NHS for alendronate.

**
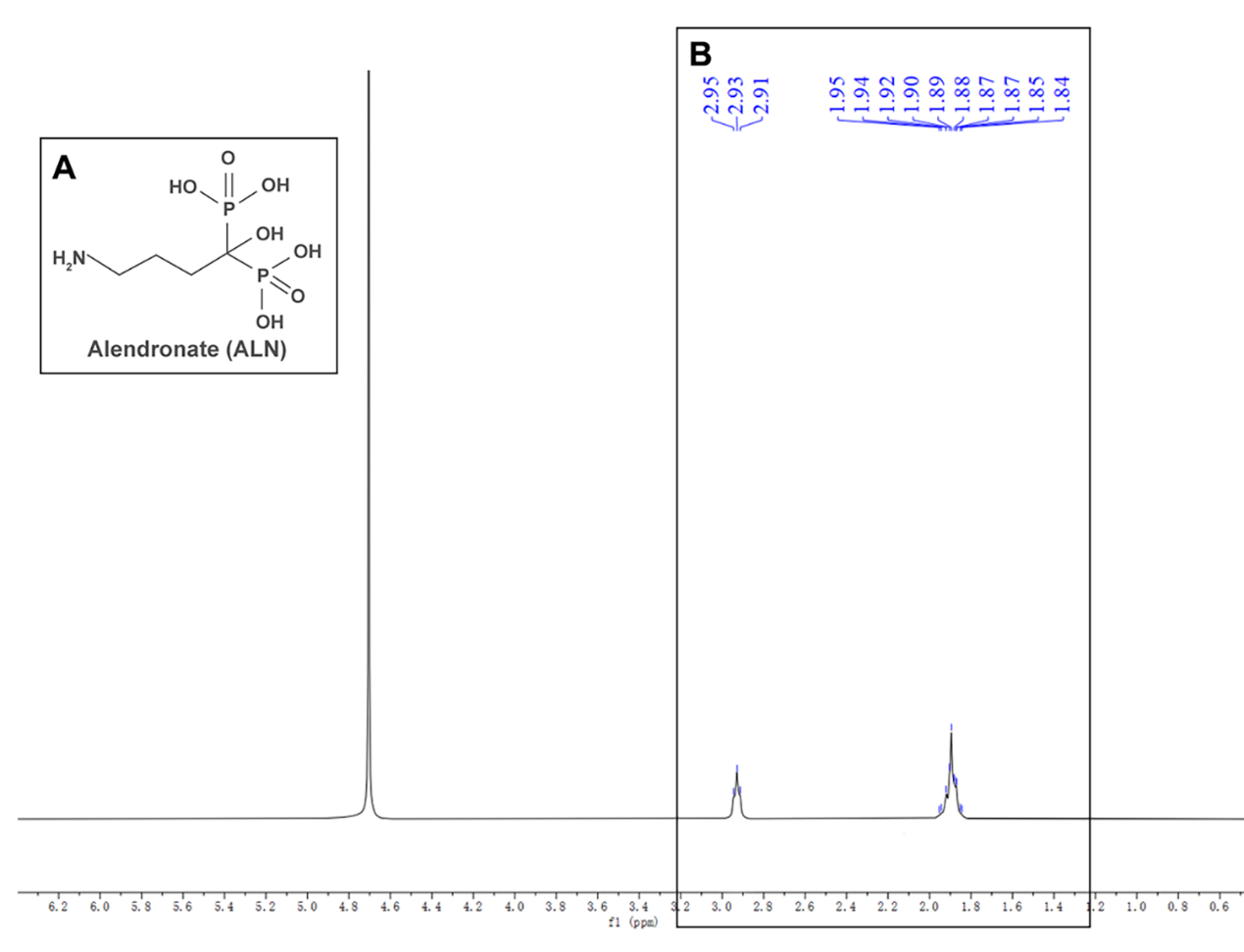
 Additional file 1: Figure S2. ^1^H-NMR spectroscopy result of ALN.**

(A). Chemical structure of ALN. (B). ^1^H-NMR results of ALN in the range of 1.2ppm to 3.2 ppm. The characteristic signal at 1.88 and 2.93 ppm corresponding to CH_2_ protons.


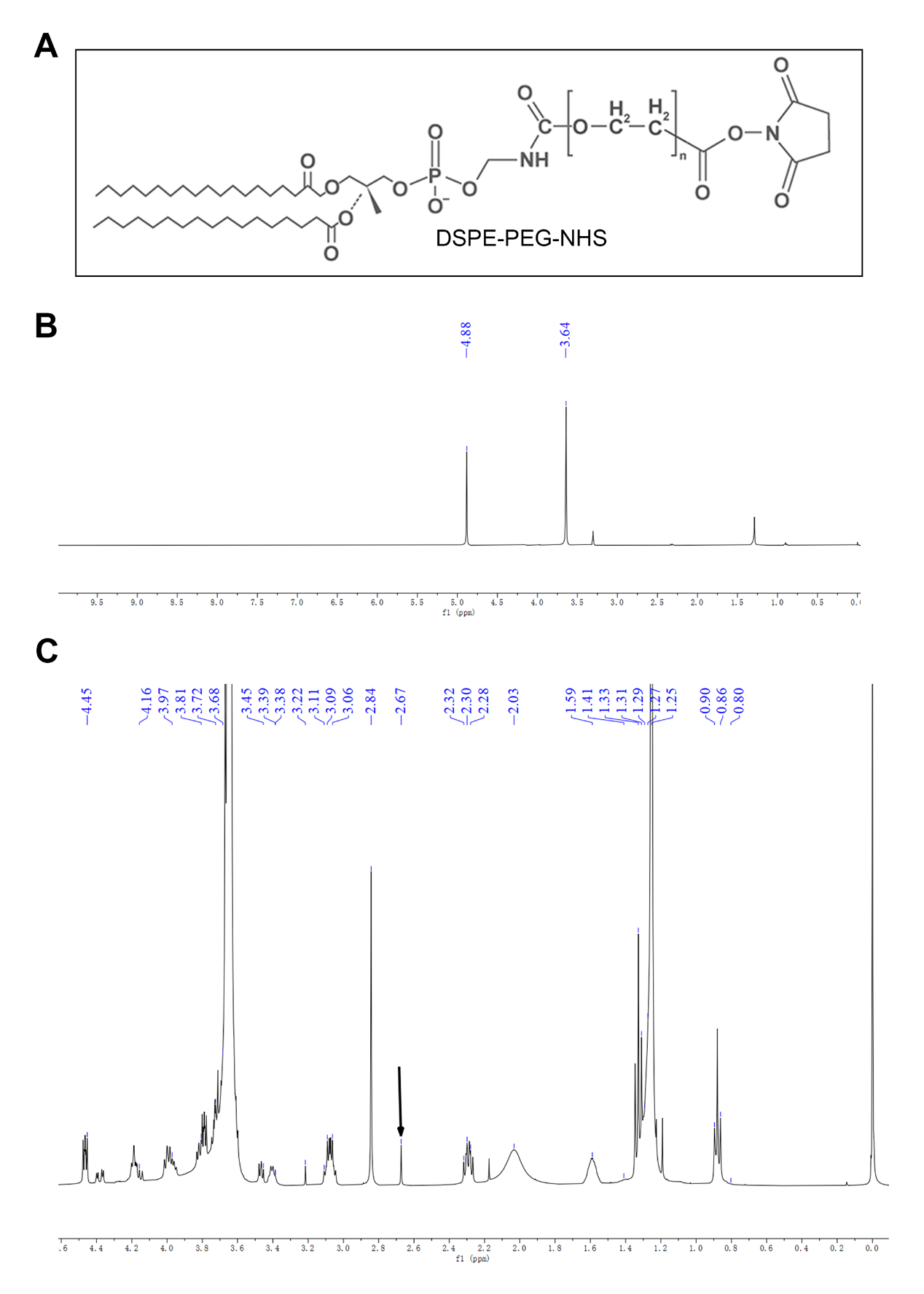


**Additional file 1: Figure S3. ^1^H-NMR spectroscopy result of DSPE-PEG-NHS.**

(A). Chemical structure of DSPE-PEG-NHS. (B). ^1^H-NMR results of DSPE-PEG-NHS in the range of 0 ppm to 10 ppm, evidencing the main peak of PEG chain (3.64 ppm). (C). ^1^H-NMR results of DSPE-PEG-NHS in the range of 0 ppm to 4.6 ppm, evidencing the carbon lateral chains (1.25 ppm), terminal methyl groups (0.80 ppm) and NHS (2.67 ppm, arrow).


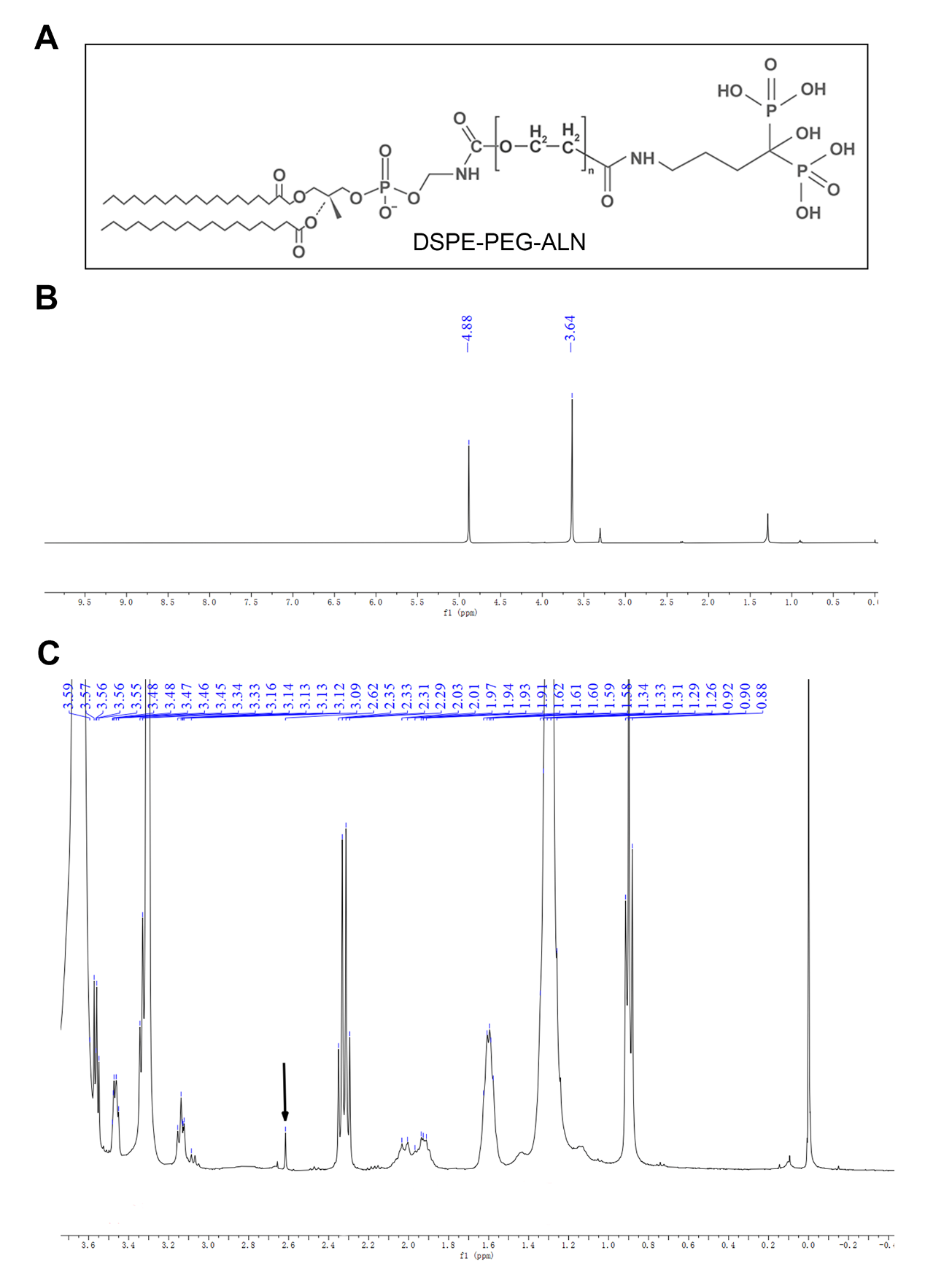


**Additional file 1: Figure S4. ^1^H-NMR spectroscopy result of DSPE-PEG-ALN.**

(A). Chemical structure of DSPE-PEG-ALN. (B). ^1^H-NMR results of DSPE-PEG-ALN in the range of 0 ppm to 10 ppm, evidencing the main peak of PEG chain (3.64 ppm). (C). ^1^H-NMR results of DSPE-PEG-ALN in the range of 0 ppm to 3.7 ppm showed a reduction of NHS group signal (2.67 ppm) and the appearance of ALN characteristic signal (1.98 and 3.13 ppm).


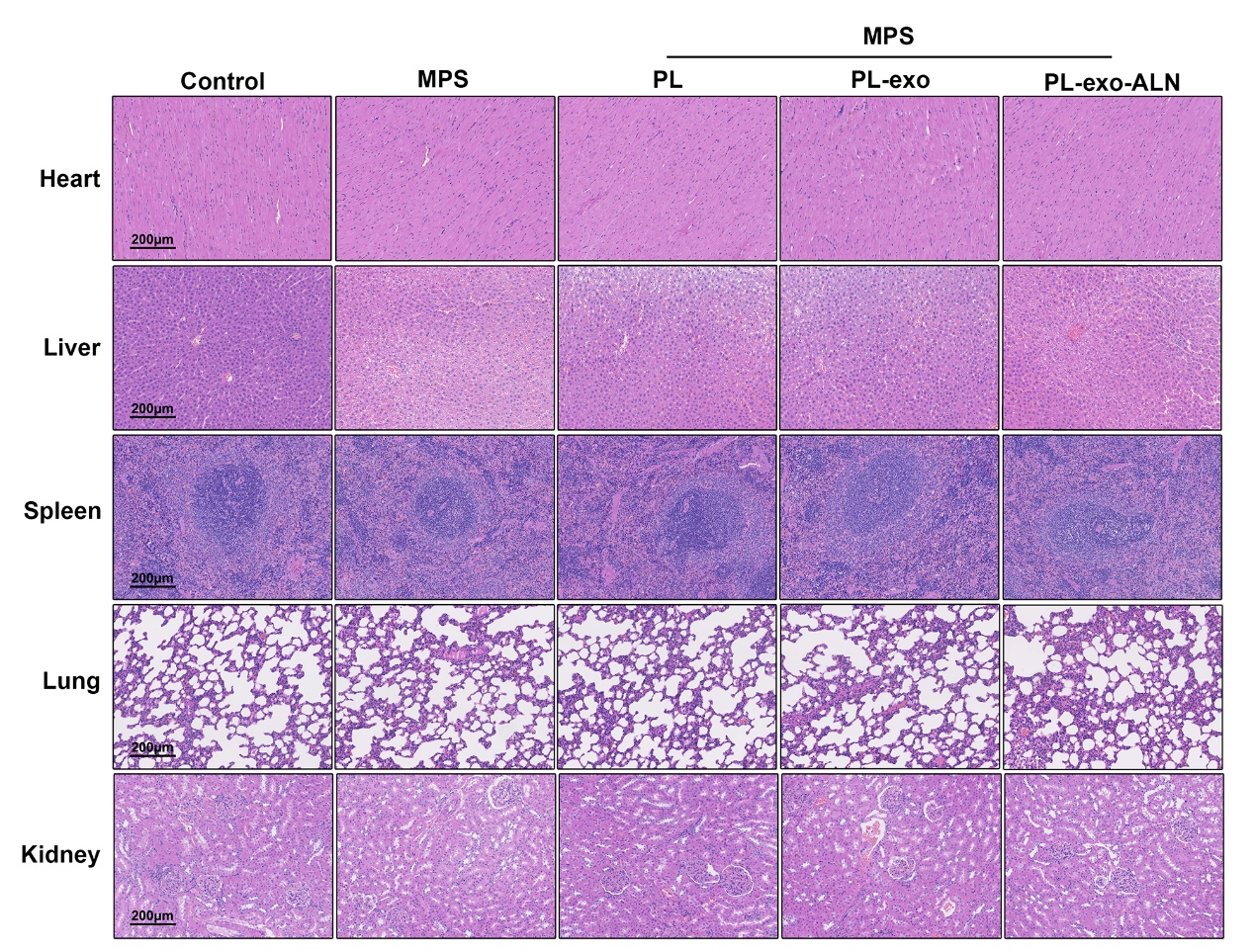


**Additional file 1: Figure S5. H&E staining of** **heart, liver, spleen, lung and kidney in five groups.**

**
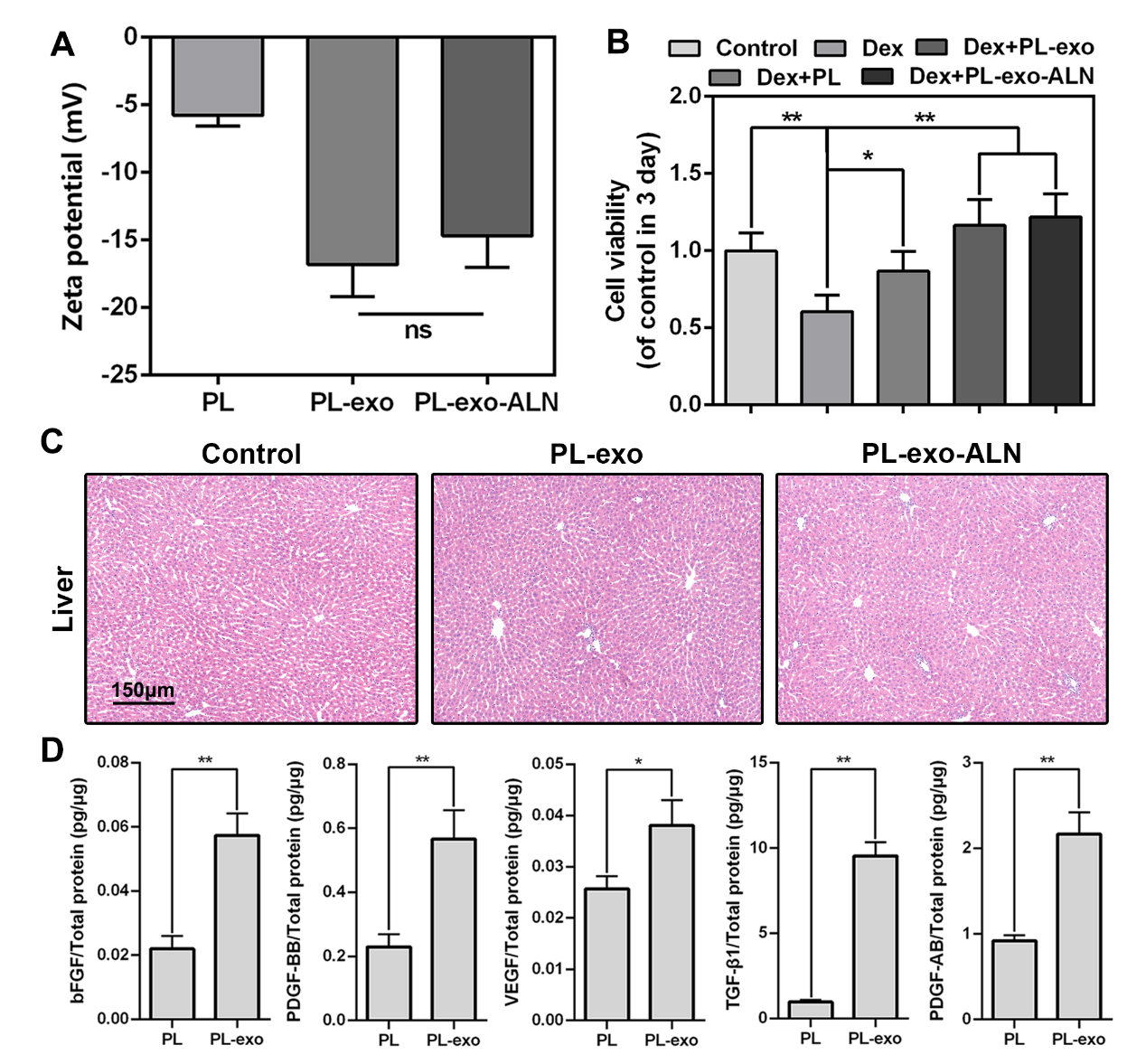
**

**Additional file 1: Figure S6.** (A) Zeta potential value of PL, PL-exo and PL-exo-ALN. (B) The cell viability of BMSCs treated as above was detected by CCK-8. (C) H&E staining of liver in the above groups. (D) The osteogenesis- and angiogenesis-associated growth factors in PL and PL-exo was determined by ELISA. All results are presented as the means ± SDs, *P < 0.05, **P < 0.01.

**
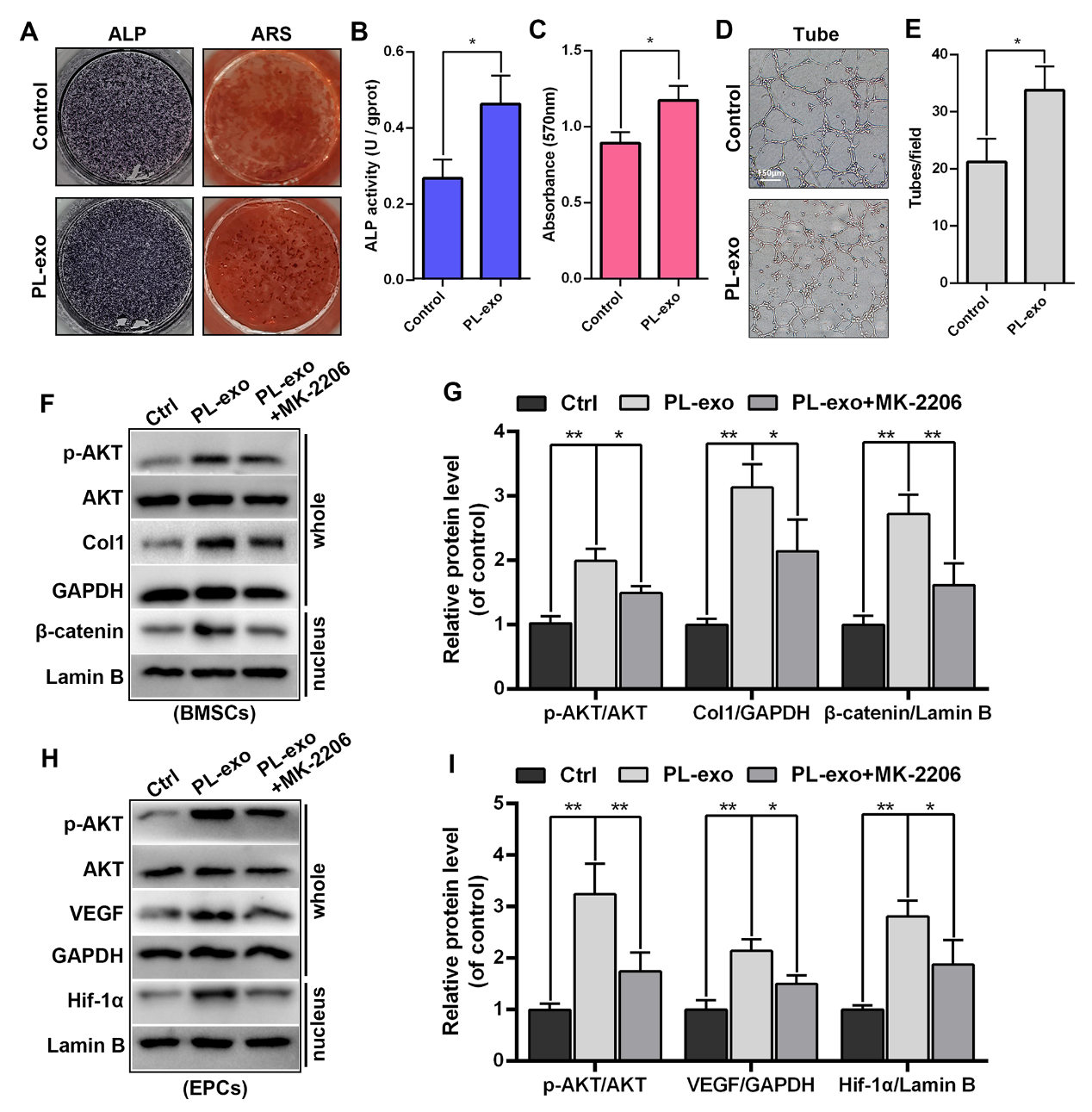
**

**Additional file 1: Figure S7.** **Effects of PL-exo on osteogenic and angiogenic differentiation of BMSCs and EPCs.**

(A-C) Early osteogenic differentiation was determined by ALP staining and ALP activity assays after 5 days of induction. Late osteogenic differentiation was determined by Alizarin Red staining and the calcium deposition was quantified by measuring the optical density, after 14 days of induction. (D-E) *In vitro* tube formation assay of EPCs treated as indicated (scale bar: 150 μm). (F-G) The expression levels of p-AKT, AKT, Col1 and β-catenin in BMSCs treated as indicated for 36 h. Exosomes (50 µg/mL) and MK-2206 (5μM, a selective inhibitor of AKT) were added to the related medium in the experimental groups, while the control group was cultured in osteogenic induction medium without any addition. (H-I) The expression levels of p-AKT, AKT, VEGF and Hif-α in EPCs treated as indicated for 24 h. Exosomes (50 µg/mL) and MK-2206 (5μM) were added to the related medium in the experimental groups, while the control group was cultured in EGM-2 complete medium without any addition. All results are presented as the means ± SDs, ^*^P < 0.05, ^**^P < 0.01.

**
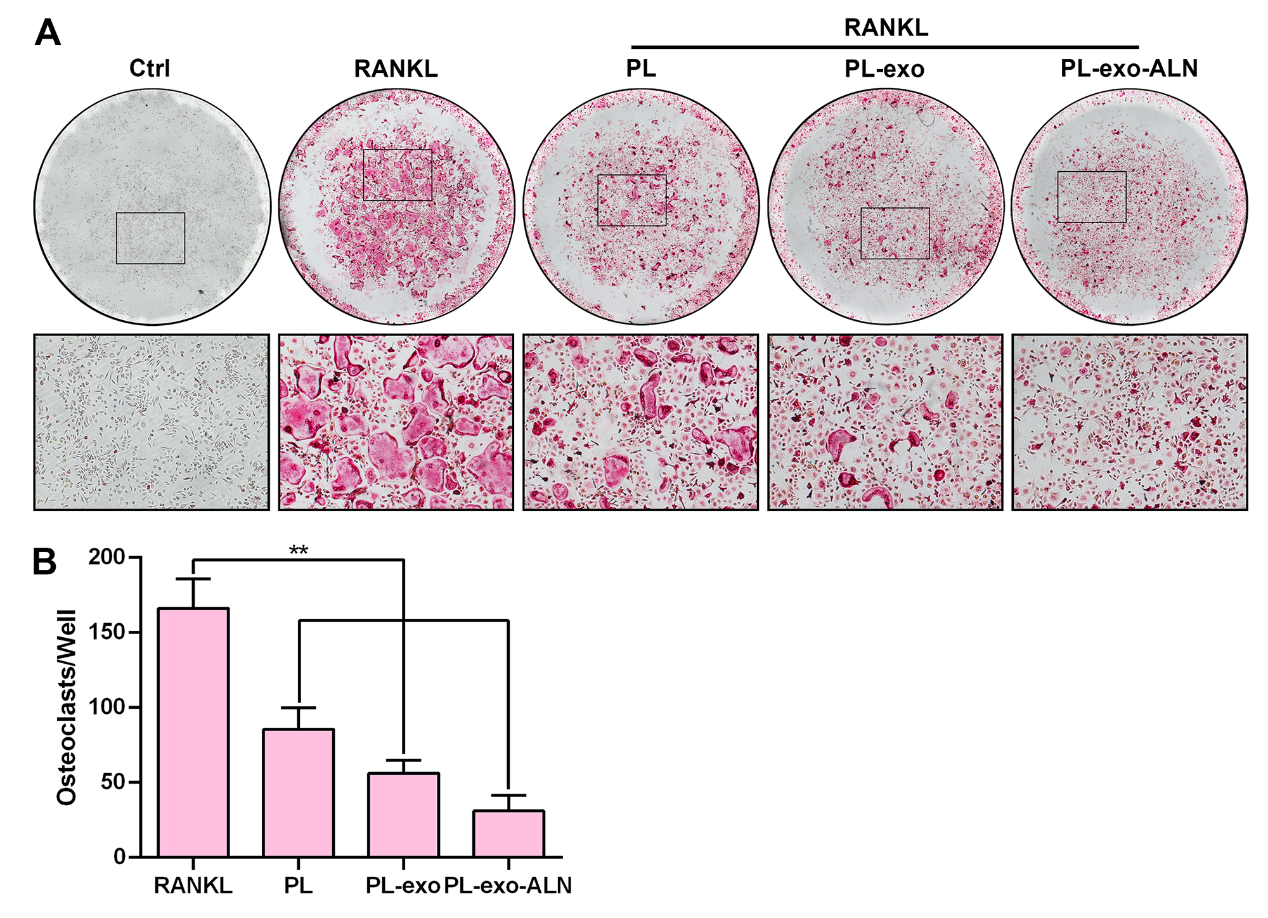
**

**Additional file 1: Figure S8. Effects of PL, PL-exo and PL-exo-ALN on RANKL-induced osteoclastogenesis in BMMs.** (A) Representative images of TRAcP-stained osteoclasts after treated with PL, PL-exo and PL-exo-ALN. (B) The quantitative analysis of TRAcP-positive multinucleated cells (>3 nuclei) per well (96-well plate). All results are presented as the means ± SDs, **P < 0.01.

**2. Additional file 1: Table**

**Additional file 1: Table S1. Post hoc power analyses of the different experiments (Unpaired t-tests).**

| **Item** | **Figure** | **n** | **Analysis method** | **Mean1±SD** | **Mean2±SD** | **Effect size d** | **Power(1-β)** |
| --- | --- | --- | --- | --- | --- | --- | --- |
| HAp binding | 2C | 3 | Unpaired t-tests | 21.210±2.975 | 78.090±6.156 | 11.7652 | 100.0% |
| ELISA (bFGF) | S6D | 3 | Unpaired t-tests | 0.022±0.004 | 0.0574±0.0069 | 6.2963 | 99.98% |
| ELISA (PDGF-BB) | S6D | 3 | Unpaired t-tests | 0.230±0.040 | 0.567±0.0902 | 4.8262 | 98.94% |
| ELISA (VEGF) | S6D | 3 | Unpaired t-tests | 0.0257±0.0025 | 0.0380±0.0050 | 3.1477 | 81.79% |
| ELISA (TGF-β1) | S6D | 3 | Unpaired t-tests | 0.970±0.121 | 9.533±0.802 | 14.9283 | 100.0% |
| ELISA (PDGF-AB) | S6D | 3 | Unpaired t-tests | 0.920±0.066 | 2.167±0.2517 | 6.7802 | 99.99% |
| ALP activity | S7B | 3 | Unpaired t-tests | 0.2675±0.0489 | 0.4626±0.0752 | 3.0764 | 80.13% |
| ARS (absorbance) | S7C | 3 | Unpaired t-tests | 0.8922±0.0728 | 1.173±0.09609 | 3.2949 | 84.91% |
| Tubes formation | S7E | 3 | Unpaired t-tests | 21.18±4.081 | 33.73±4.222 | 3.0226 | 78.82% |

**Additional file 1: Table S2. Post hoc power analyses of the different experiments (One-way ANOVA).**

| **Item** | **Figure** | **n** | **Analysis method** | **SS_A_** | **SS_T_** | **Partial η^2^** | **Effect size f** | **Power(1-β)** |
| --- | --- | --- | --- | --- | --- | --- | --- | --- |
| ALP activity | 3B | 3 | One-way ANOVA | 0.04129 | 0.04372 | 0.9444 | 4.1221 | 100.00% |
| ARS (absorbance) | 3D | 3 | One-way ANOVA | 0.6756 | 0.6972 | 0.9690 | 5.5927 | 100.00% |
| WB (Col-I) | 3F | 3 | One-way ANOVA | 1.2070 | 1.2370 | 0.9757 | 6.3430 | 100.00% |
| WB (RUNX-2) | 3F | 3 | One-way ANOVA | 1.0430 | 1.0570 | 0.9868 | 8.6313 | 100.00% |
| WB (OCN) | 3F | 3 | One-way ANOVA | 1.6310 | 1.6460 | 0.9909 | 10.4275 | 100.00% |
| Tubes formation | 4C | 3 | One-way ANOVA | 716.90 | 752.90 | 0.9522 | 4.4625 | 100.00% |
| Migrating cells | 4D | 3 | One-way ANOVA | 12167.0 | 12579.0 | 0.9672 | 5.4343 | 100.00% |
| WB (p-PDGF) | 4F | 3 | One-way ANOVA | 1.7880 | 1.9100 | 0.9361 | 3.8283 | 100.00% |
| WB (p-FAK) | 4F | 3 | One-way ANOVA | 1.1040 | 1.1300 | 0.9770 | 6.5163 | 100.00% |
| WB (PDGF-BB) | 5B | 3 | One-way ANOVA | 1.2280 | 1.3930 | 0.8816 | 2.7281 | 100.00% |
| WB (VEGF) | 5B | 3 | One-way ANOVA | 2.9230 | 3.2100 | 0.9106 | 3.1913 | 100.00% |
| WB (BMP-2) | 5D | 3 | One-way ANOVA | 3.6260 | 3.9560 | 0.9166 | 3.3148 | 100.00% |
| WB (OPG) | 5D | 3 | One-way ANOVA | 1.8990 | 2.0670 | 0.9187 | 3.3621 | 100.00% |
| ALP activity | 5G | 3 | One-way ANOVA | 0.0096 | 0.0106 | 0.9071 | 3.1248 | 100.00% |
| ARS (absorbance) | 5H | 3 | One-way ANOVA | 0.1493 | 0.1565 | 0.9540 | 4.5537 | 100.00% |
| WB (Col-I) | 5J | 3 | One-way ANOVA | 7.3540 | 7.5490 | 0.9742 | 6.1411 | 100.00% |
| WB (RUNX-2) | 5J | 3 | One-way ANOVA | 1.8670 | 1.9680 | 0.9487 | 4.2994 | 100.00% |
| WB (OCN) | 5J | 3 | One-way ANOVA | 3.9670 | 4.1670 | 0.9520 | 4.4537 | 100.00% |
| Tubes formation | 5M | 3 | One-way ANOVA | 26.2500 | 32.2500 | 0.8140 | 2.0917 | 99.85% |
| Migrating cells | 5N | 3 | One-way ANOVA | 1875.0 | 2030.0 | 0.9236 | 3.4780 | 100.00% |
| WB (p-PDGF) | 5P | 3 | One-way ANOVA | 0.0277 | 0.0927 | 0.2984 | 0.6522 | 29.28% |
| WB (p-FAK) | 5P | 3 | One-way ANOVA | 0.6675 | 0.8137 | 0.8203 | 2.1367 | 99.89% |
| MicroCT (BMD) | 6B | 5 | One-way ANOVA | 83487.0 | 93694.0 | 0.8911 | 2.8600 | 100.00% |
| MicroCT (BV/TV) | 6B | 5 | One-way ANOVA | 7097.0 | 7516.0 | 0.9443 | 4.1156 | 100.00% |
| MicroCT (Tb.N) | 6B | 5 | One-way ANOVA | 17.030 | 19.890 | 0.8562 | 2.4402 | 100.00% |
| MicroCT (Tb.Sp) | 6B | 5 | One-way ANOVA | 1.3560 | 1.4830 | 0.9144 | 3.2676 | 100.00% |
| MicroCT (Tb.Th) | 6B | 5 | One-way ANOVA | 0.0522 | 0.0638 | 0.8186 | 2.1245 | 100.00% |
| H&E (fat tissue) | 7B | 5 | One-way ANOVA | 3417.0 | 3650.0 | 0.9362 | 3.8295 | 100.00% |
| IHC (Col-I) | 7D | 5 | One-way ANOVA | 1.1290 | 1.1800 | 0.9568 | 4.7050 | 100.00% |
| IHC (OCN) | 7F | 5 | One-way ANOVA | 0.4729 | 0.5261 | 0.8989 | 2.9815 | 100.00% |
| Trap (Oc.S/BS) | 7H | 5 | One-way ANOVA | 1424.0 | 1555.0 | 0.9158 | 3.2970 | 100.00% |
| MicroCT (vessel volume) | 8B | 3 | One-way ANOVA | 18.5400 | 19.080 | 0.9717 | 5.8595 | 100.00% |
| IHC (VEGF) | 8D | 5 | One-way ANOVA | 0.8222 | 0.8842 | 0.9299 | 3.6416 | 100.00% |
| IF (Type H vessel) | 8F | 3 | One-way ANOVA | 0.7819 | 0.8266 | 0.9459 | 4.1824 | 100.00% |
| Zeta potential | S6A | 3 | One-way ANOVA | 343.200 | 390.20 | 0.8795 | 2.7022 | 99.98% |
| Cell viability | S6B | 3 | One-way ANOVA | 1.2190 | 1.5820 | 0.7705 | 1.8325 | 99.74% |
| Osteoclasts/Well | S8B | 3 | One-way ANOVA | 31060.0 | 32590.0 | 0.9531 | 4.5055 | 100.00% |
| WB (p-AKT) | S7G | 3 | One-way ANOVA | 1.4190 | 1.5400 | 0.9214 | 3.4245 | 100.00% |
| WB (Col1) | S7G | 3 | One-way ANOVA | 6.8350 | 7.6010 | 0.8992 | 2.9871 | 100.00% |
| WB (β-catenin) | S7G | 3 | One-way ANOVA | 4.5520 | 5.0020 | 0.9100 | 3.1805 | 100.00% |
| WB (p-AKT) | S7I | 3 | One-way ANOVA | 7.8470 | 8.8340 | 0.8883 | 2.8200 | 99.99% |
| WB (VEGF) | S7I | 3 | One-way ANOVA | 1.969 | 2.201 | 0.8946 | 2.9134 | 100.00% |
| WB (Hif-α) | S7I | 3 | One-way ANOVA | 4.882 | 5.536 | 0.8819 | 2.7327 | 99.99% |

**SS_A_ is called variation between groups and represents the sum of the squares of the mean differences between each group and the population. SS_T_ is termed total variation and represents the sum of squares of deviations from mean. Partial η^2^=SS_A_/SS_T_**
